# Independent and Interactive Associations of Encopresis and Attention-Deficit/Hyperactivity Disorder in Children on Emotional–Behavioral Symptoms, Executive Functioning, and Avoidant/Restrictive Food Intake–Related Symptoms

**DOI:** 10.1007/s10802-026-01463-w

**Published:** 2026-04-21

**Authors:** Makbule Esen Öksüzoğlu, Hande Günal Okumuş, Nagihan Özer, Hilal Subaşı

**Affiliations:** 1 Present Address: Department of Child and Adolescent Psychiatry, Kastamonu University Faculty of Medicine, Kastamonu, Turkey; 2 Department of Child and Adolescent Psychiatry, Uşak Training and Research Hospital, Uşak, Turkey; 3 Department of Child and Adolescent Psychiatry, Şırnak State Hospital, Şırnak, Turkey; 4 Department of Child and Adolescent Psychiatry, Kahramanmaraş City Hospital, Kahramanmaraş, Turkey

**Keywords:** Encopresis, Attention-Deficit/Hyperactivity Disorder, Executive functioning, Interaction effects, Emotional symptoms

## Abstract

In this multicenter, cross-sectional case–control study, we examined the independent and interactive associations of encopresis and attention-deficit/hyperactivity disorder (ADHD) with emotional-behavioral symptoms, executive functioning, and avoidant/restrictive food intake-related symptoms in children. The sample included 148 children aged 5–10 years assigned to four groups: healthy controls (*n* = 31), ADHD-only (*n* = 60), encopresis-only (*n* = 31), and encopresis plus ADHD (*n* = 26). Diagnoses were established using clinician-administered semi-structured interviews, and parent-report measures assessed emotional-behavioral symptoms, executive functioning, and eating-related symptoms. Across analyses, impairment followed a graded pattern, with the encopresis plus ADHD group showing the highest level of impairment (*H* = 89.84, *p* < 0.001). Emotional symptoms and internalizing problems were most pronounced in the encopresis-only and comorbid groups (*p* < 0.001). Fear-based eating behaviors were significantly elevated in the comorbid group (*H* = 25.00, *p* < 0.001). Executive functioning impairments also followed a graded pattern, with the greatest dysfunction in the comorbid group (*p* < 0.001). After additional adjustment for Clinical Global Impression-Severity, encopresis × ADHD interaction effects remained significant for emotional symptoms (*β* = 3.227, *p* = 0.011) and fear-based eating (*β* = 3.196, *p* = 0.033). Children with co-occurring encopresis and ADHD showed the most severe cross-sectional clinical profile, highlighting the importance of assessing emotional symptoms, executive functioning, and fear-based eating in this subgroup.

**Supplementary Information:**

The online version contains supplementary material available at 10.1007/s10802-026-01463-w.

## Introduction

Encopresis (ENC) is a childhood elimination disorder characterized by recurrent fecal soiling beginning at age four, following the exclusion of organic causes and after the typical developmental period for bowel control (American Psychiatric Association, 2013). The prevalence of ENC is estimated at 1–3% among preschool-aged children and declines throughout later childhood and adolescence, with a spontaneous remission rate of approximately 15% per year (Rey & Omigbodun, 2015). Although ENC was previously conceptualized as a gastrointestinal disorder primarily linked to constipation and functional defecation issues, recent evidence indicates that it is a multidimensional condition involving behavioral, emotional, and neurodevelopmental factors. Compared to their peers, children with ENC frequently experience psychosocial challenges, including attention difficulties, behavioral dysregulation, social impairments, and reduced self-esteem (Cox et al., 2002; Gizli Çoban et al., 2021; Kuizenga-Wessel et al., 2018; Yilmaz et al., 2014). Meta-analytic studies have identified small-to-moderate impairments in these domains, with the most pronounced effects observed in attention-related difficulties (Aymerich et al., 2023). These findings suggest that ENC may represent a broader self-regulatory dysfunction rather than a purely somatic disorder.

Attention-Deficit/Hyperactivity Disorder (ADHD) is a prevalent neurodevelopmental disorder in childhood, characterized by persistent patterns of inattention, hyperactivity, impulsivity, and deficits in executive and behavioral regulation (Polanczyk et al., 2015). Epidemiological and clinical evidence increasingly supports significant associations between ADHD and elimination-related difficulties. A large population-based study of 742,939 individuals estimated ADHD prevalence at 4.4% and found that constipation was significantly more common among individuals with ADHD compared to the general population (4.1% vs. 1.5%) (McKeown et al., 2013). Proposed mechanisms underlying this association include reduced awareness of interoceptive cues due to inattention, impulsivity-driven postponement of toileting in the presence of competing stimuli, and executive dysfunction, particularly impairments in inhibitory control, planning, working memory, and self-monitoring, which may disrupt the establishment and maintenance of consistent toileting routines (Allah et al., 2023; Shreeram et al., 2009; Steward et al., 2017; von Gontard et al., 2011). Epidemiological research further supports this perspective by identifying associations between ADHD and maladaptive toileting behaviors, such as holding maneuvers, intermittency, and excessive stool retention, which may contribute to the development or persistence of ENC (Liu & Zhang, 2022).

Encopresis and ADHD co-occur at rates that are significantly higher than would be expected by chance, with comorbidity estimates ranging from approximately 10% to 40% (Rey & Omigbodun, 2015). Children with functional defecation disorders demonstrate elevated rates of ADHD, and, conversely, ADHD is associated with an increased risk of encopresis and related elimination problems (Kuizenga-Wessel et al., 2018). Clinical samples report ADHD in up to 40–53% of children with ENC, and population-based studies also reveal robust associations between these conditions (Allah et al., 2023; Liu & Zhang, 2022; Niemczyk et al., 2015; Shreeram et al., 2009). Collectively, these findings underscore the developmental and clinical significance of the comorbidity between ADHD and ENC.

The clinical overlap between ENC and ADHD may be conceptualized across multiple domains of self-regulation, including executive functioning, internalizing symptoms, sensory processing, sleep-related regulation, and eating-related behaviors (Aymerich et al., 2023). In the present study, Avoidant/Restrictive Food Intake Disorder (ARFID) related symptoms were included because they represent a clinically proximal and relatively understudied candidate domain at the intersection of neurodevelopmental features and bowel symptoms. Specifically, emerging evidence suggests that children with ADHD may show elevated selective or ARFID related symptoms, while pediatric studies have also reported bidirectional associations between selective eating and functional constipation (Kaşak et al., 2025). In addition, findings from neurodevelopmental samples suggest that food selectivity may contribute to constipation-related symptoms in at least some children (Harris et al., 2021). Sensory sensitivity, fear of aversive consequences, and interoceptive processes may provide one possible mechanistic bridge between avoidant eating and bowel-related discomfort, although these pathways remain tentative and have not been directly established in children with encopresis (Beaudry-Bellefeuille & Lane, 2017; Beaudry-Bellefeuille et al., 2019; Thomas et al., 2017). Accordingly, we approached ARFID-related symptoms as one exploratory domain within a broader multidomain self-regulatory framework.

Restrictive and selective eating behaviors may represent an additional, yet underexplored, domain associated with both neurodevelopmental dysregulation and elimination difficulties. Within a neurodevelopmental framework, these eating behaviors are closely associated with executive functioning deficits, particularly impairments in cognitive flexibility and inhibitory control. Such deficits may impede the regulation of rigid or avoidant eating patterns, including food refusal, and similar regulatory demands are required for consistent toileting behaviors. The phenotype of Avoidant/Restrictive Food Intake Disorder (ARFID) demonstrates substantial overlap with neurodevelopmental conditions such as ADHD and autism spectrum disorder, both of which are characterized by executive dysfunction (Bourne et al., 2022; Kaşak et al., 2025; Nyholmer et al., 2025; Thomas et al., 2017). Additionally, Zafari et al. identified low intake of dietary fiber, fruits, and vegetables as a significant risk factor for constipation, and similar dietary patterns are frequently observed in children with selective eating (Zafari et al., 2021). Consequently, difficulties in regulating eating behavior may co-occur with constipation and ENC as part of a broader self-regulatory dysfunction, rather than as an isolated feeding problem. However, eating-related traits have not yet been systematically investigated in children with encopresis.

Although epidemiological evidence for comorbidity is robust, the current literature provides limited insight into the combined clinical impact of ENC and ADHD. Most studies have examined ENC and ADHD separately or have focused primarily on prevalence estimates, offering little information on comorbid clinical profiles (Gizli Çoban et al., 2021). Furthermore, the predominant use of two-group comparisons limits the ability to determine whether co-occurrence is characterized primarily by overlapping clinical features, higher overall symptom levels, or statistically detectable interaction patterns. Therefore, there is a clear need for cross-sectional comparative studies that include healthy controls (HC), ADHD-only, ENC-only, and comorbid ENC + ADHD groups, combined with interaction-based analytic approaches, to disentangle the independent and joint associations of ENC and ADHD across multiple functional domains.

To address these gaps, the present multicenter cross-sectional case–control study aimed to characterize the emotional, behavioral, executive, and eating-related profiles associated with ENC and ADHD, both individually and in combination, across four clinically defined groups (HC, ADHD-only, ENC-only, and ENC + ADHD). Emotional and behavioral functioning were assessed with explicit differentiation between internalizing and externalizing symptoms, together with executive functioning and eating-related behaviors. We then applied a 2 × 2 factorial analytic framework in the regression models to examine whether the co-occurrence of ENC and ADHD was better characterized by independent associations, additive burden, or statistical interaction (ENC × ADHD). The study tested the following hypotheses: (1) Children with comorbid ENC and ADHD would exhibit greater impairments in executive functioning, higher levels of internalizing and externalizing symptoms, more severe ADHD-related symptoms, and ARFID-related symptoms compared to children with ADHD alone. (2) ADHD was expected to be the primary determinant of impairments in executive functioning and the core symptoms of ADHD, including inattention, hyperactivity, and impulsivity, while the specific clinical domains associated with encopresis would be explored. In addition, as a secondary, clinically oriented analysis, we explored whether children with ENC differed by retentive versus non-retentive subtype with respect to selected clinical characteristics and symptom severity.

## Methods

### Participants

This multicenter study used a cross-sectional, case–control design. Participants were recruited from the child and adolescent psychiatry outpatient clinics of four hospital-based centers across different geographical regions of Türkiye: Uşak Training and Research Hospital in the Aegean region, Kastamonu Training and Research Hospital in the Black Sea region, Şırnak State Hospital in the Southeastern Anatolia region, and Kahramanmaraş City Hospital in the Mediterranean region. These clinics serve a heterogeneous outpatient pediatric population, including children presenting with suspected psychiatric symptoms, developmental concerns, behavioral difficulties, and situational problems for which families seek counseling or guidance. Although participants were classified into four clinically defined groups, no experimental manipulation or random assignment was involved. Participants were recruited consecutively from children aged 5 to 10 years who presented for their first psychiatric evaluation at the Child and Adolescent Psychiatry outpatient clinics of four hospitals between October 1, 2025, and January 25, 2026.

Based on clinical evaluation, participants were classified into to one of four predefined groups: (1) healthy controls (HC), (2) ADHD, (3) ENC and (4) ENC + ADHD group. Children with ENC were further classified as having retentive or non-retentive encopresis according to Rome IV criteria.

General inclusion criteria for all participants were: (a) age between 5 and 10 years, (b) voluntary participation, (c) availability of a literate parent able to complete parent-report questionnaires, (d) written informed consent from parents and verbal assent from children, and (e) absence of prior psychotropic medication use.

Group-specific inclusion criteria were as follows:

I. ADHD-only group: diagnosis of ADHD according to DSM-5 criteria;
II. ENC-only group: diagnosis of ENC without comorbid ADHD;
III. ENC + ADHD group: diagnosis of ADHD with comorbid ENC;
IV. Healthy control group: absence of any current or lifetime psychiatric diagnosis.

Only children undergoing their first psychiatric evaluation and not yet exposed to psychotropic treatment were included. This approach was chosen to allow the assessment of eating behaviors, executive functioning, and clinical impairment before the potential modifying effects of medication. Exclusion criteria applied to all groups included the presence of autism spectrum disorder, intellectual disability, or severe language disorder; chronic neurological, endocrine, or gastrointestinal medical conditions; any major psychiatric disorder other than ADHD. Cases with incomplete assessment data or withdrawal after enrollment were treated as attrition and excluded from the final analytic sample rather than considered exclusion criteria. During the recruitment process, children who did not meet eligibility criteria were excluded before final enrollment. Specifically, 2 children were excluded because they were outside the predefined age range and were 11 years old. Three children were excluded due to autism spectrum disorder. Among children evaluated for the ADHD groups, 18 were excluded because of comorbid psychiatric disorders and 23 because of a history of stimulant treatment, although treatment had been discontinued approximately 1 to 2 years earlier. Among children evaluated for the encopresis groups, 2 were excluded because of meningomyelocele and 4 because of other comorbid medical conditions.

Healthy controls were selected from children presenting to the participating child and adolescent psychiatry outpatient clinics for counseling purposes, including developmental guidance and support for acute situational difficulties such as family or peer-related problems. To ensure their suitability as controls, all underwent a semi-structured psychiatric assessment and were included only if no current or lifetime psychiatric disorder was identified.

### Procedure

This study was approved by the Non-Interventional Clinical Research Ethics Committee of Uşak University (No: 846–846-04, Date: 25/09/2025) and conducted in accordance with the Declaration of Helsinki. Parents provided written informed consent, and children provided verbal assent after receiving a full explanation of the study procedures.

Psychiatric diagnoses were confirmed through clinician-administered semi-structured interviews conducted by trained child and adolescent psychiatrists. After psychiatric diagnoses were confirmed, parents completed a structured sociodemographic form developed by the research team. This form collected information on the child’s age, sex, school grade, and relevant family characteristics. Toilet-related characteristics and bowel habits were systematically evaluated through parent reports and clinical interviews. Parents were asked about toilet training status, age at first toilet training attempt, total duration of toilet training, and the number of failed attempts. Stool consistency was assessed with the Bristol Stool Form Scale, a validated visual classification system that categorizes stool form into seven types based on consistency and appearance (Lewis & Heaton, 1997). Constipation-related features were evaluated according to Rome IV criteria, including stool frequency, retentive posturing or stool withholding, painful or hard bowel movements, passage of large-diameter stools that may obstruct the toilet, and the presence of a fecal mass in the rectum.

The frequency of fecal incontinence episodes was recorded over the past 3 months (≥ 1 episode per month) and over the past month (≥ 1 episode per week). Encopresis subtype (retentive vs. non-retentive) and onset type (primary vs. secondary) were determined through clinical evaluation. For children who had achieved a dry period, the duration (in months) was recorded.

Additional lifestyle variables relevant to bowel function were assessed. These included weekly bowel movement frequency, daily water intake (glasses per day), screen time (hours per day), and physical activity levels, measured by both the number of days per week and daily duration (minutes per day) of moderate-to-vigorous activity.

Anthropometric measurements, including height and weight, were obtained by trained clinical staff during the outpatient visit. Body mass index (BMI, kg/m^2^) was calculated and converted to age- and sex-adjusted BMI z-scores using the 2007 World Health Organization growth reference for school-aged children and adolescents (de Onis et al., 2007).

Parents completed the Conners’ Parent Rating Scale-Revised: Short Form (CPRS-R:S), the Strengths and Difficulties Questionnaire (SDQ), the Behavior Rating Inventory of Executive Function—Parent Form (BRIEF), and the Nine-Item Avoidant/Restrictive Food Intake Disorder Screen (NIAS). Researchers rated the severity of ENC using the Clinical Global Impression–Severity (CGI-S) scale.

### Psychometric Measures

#### The Kiddie Schedule for Affective Disorders and Schizophrenia for School-Age Children—Present and Lifetime Version DSM–5 (K-SADS-PL)

This semi-structured interview is designed to detect current and lifetime psychopathologies in children and adolescents (Kaufman et al., 1997), has been adapted for DSM-5, and is validated for use in Turkish pediatric populations (Unal et al., 2019). The K-SADS-PL was administered to both children and their parents to identify comorbid psychiatric conditions in the clinical groups and to confirm the absence of psychiatric diagnoses in the healthy control group.

#### Conners’ Parent Rating Scale-Revised: Short Form (CPRS-R:S)

The Conners’ Parent Rating Scale–Revised: Short Form (CPRS-R:S) is a 27-item parent-report questionnaire developed to assess attention deficit, hyperactivity/impulsivity, oppositional behavior, and related behavioral difficulties in children (Conners et al., 1998). Items are rated on a 4-point Likert scale ranging from 0 (never) to 3 (very often). The scale yields four subscales: Cognitive Problems/Inattention (6 items), Hyperactivity (6 items), Oppositional Behavior (3 items), and the ADHD Index (12 items), with higher scores indicating greater symptom severity. The Turkish adaptation of the scale, along with its validity and reliability, was established by Kaner et al. (Kaner et al., 2013). In this study, Cronbach’s alpha was 0.955.

#### Strengths and Difficulties Questionnaire (SDQ)

The Strengths and Difficulties Questionnaire (SDQ) is a 25-item behavioral screening instrument designed to assess emotional and behavioral functioning in children and adolescents aged 4–16 years (Goodman, 1997). Items are rated on a 3-point Likert scale (0 = not true, 1 = somewhat true, 2 = certainly true). The SDQ comprises five subscales: Emotional Symptoms, Conduct Problems, Hyperactivity/Inattention, Peer Relationship Problems, and Prosocial Behavior. Each subscale yields a separate score, and a Total Difficulties Score is calculated by summing the first four subscales, providing an overall index of psychosocial difficulties. The SDQ has demonstrated good psychometric properties across several populations, and the Turkish validity and reliability study of the parent-report form was conducted by Dursun et al. (Dursun et al., 2019). In this study, Cronbach’s alpha was 0.759.

#### Behavior Rating Inventory of Executive Function – Parent Form (BRIEF)

The Behavior Rating Inventory of Executive Function (BRIEF) was developed by Gioia et al. (2002) to assess executive functioning in everyday settings through parent report (Gioia et al., 2002). The BRIEF evaluates executive processes as they manifest in real-life behaviors rather than performance under structured testing conditions. The scale consists of 86 items organized into eight clinical subscales: Inhibit, Shift, Emotional Control, Initiate, Working Memory, Plan/Organize, Organization of Materials, and Monitor. These subscales form two composite indices: the Behavioral Regulation Index (Inhibit, Shift, Emotional Control) and the Metacognition Index (Initiate, Working Memory, Plan/Organize, Organization of Materials, Monitor). Higher scores indicate greater executive dysfunction in daily functioning. The validity and reliability of the Turkish version of the BRIEF have been established by Erdoğan Bakar et al. (Bakar et al., 2011). In this study, Cronbach’s alpha was 0.972. As a parent-report instrument, however, the BRIEF reflects executive difficulties as observed in everyday behavior and should not be considered interchangeable with performance-based neuropsychological assessment.

#### Nine-Item Avoidant/Restrictive Food Intake Disorder Screen (NIAS)

The Nine-Item Avoidant/Restrictive Food Intake Disorder Screen (NIAS) is a brief screening instrument developed by Zickgraf and Ellis to assess symptoms of ARFID (Zickgraf & Ellis, 2018). The NIAS is available in both self-report and parent-report forms and is designed to capture core ARFID-related eating patterns across developmental stages. The scale comprises three subscales reflecting distinct ARFID dimensions: selective eating, low appetite, and fear of aversive consequences (e.g., choking or vomiting). Each item is rated on a 6-point Likert scale ranging from 1 (“not at all”) to 6 (“very much”), with higher scores indicating greater symptom severity. Established cut-off scores indicating elevated risk for ARFID are ≥ 10 for the selective eating subscale, ≥ 9 for the low appetite subscale, and ≥ 10 for the fear subscale. In the present study, the parent-report version of the NIAS was used. The parent-report Turkish version of the NIAS has demonstrated satisfactory validity and reliability in children, as reported by Öğütlü et al. (Öğütlü et al., 2024). In this study, Cronbach’s alpha was 0.786.

#### Clinical Global Impression – Severity Scale (CGI-S)

The Clinical Global Impression (CGI) scale, developed by Guy in 1976, is a clinician-rated instrument widely used to assess overall illness severity, clinical improvement, and treatment-related side effects (Guy, 1976). The scale comprises three components: the CGI–Severity (CGI-S), the CGI–Improvement (CGI-I), and the Side Effects Rating scale. The CGI-S measures current illness severity using a 7-point Likert scale, with scores ranging from 1 (normal, not at all ill) to 7 (among the most extremely ill patients). In the present study, the CGI-S was completed assessment by child and adolescent psychiatrists at each participating site. All raters had the same level of clinical specialization, and a pre-study meeting was conducted to standardize the assessment approach across centers. Ratings were based on the clinician’s overall impression of encopresis severity following the full clinical evaluation, including information obtained from both the child and parent interviews. Formal inter-rater reliability was not assessed.

### Power Analysis

An a priori power analysis was conducted to estimate the required sample size. As no previous study had examined the co-occurrence of ADHD and ENC using a directly comparable four-group design, the estimation was based on effect sizes reported in related clinical populations. Previous studies examining the effects of ADHD on executive functioning and behavioral outcomes in children have reported medium to moderately large effect sizes (Gioia et al., 2002). For the present study, a Cohen’s d of 0.65 was taken as the reference effect size. Under an equal-group approximation, this corresponds to an ANOVA effect size of approximately *f* = 0.325.

Using G*Power (version 3.1.9.4), an a priori power analysis was performed for a one-way analysis of variance (ANOVA; fixed effects, omnibus test) with four groups (HC, ADHD, ENC, and ADHD + ENC). With an alpha level of 0.05 and statistical power (1 − β) set at 0.80, the minimum required sample size was estimated to be approximately 100 participants, corresponding to about 25 participants per group.

### Statistical Analysis

All statistical analyses were conducted using IBM SPSS Statistics (version 28) and Stata (version 17). Descriptive statistics are presented as mean ± standard deviation and median (minimum–maximum) for continuous variables, and as frequencies and percentages for categorical variables.

Sociodemographic, clinical, toileting-related, behavioral, eating, and executive functioning variables were compared across the four diagnostic groups (HC, ADHD, ENC, and ENC + ADHD). Normality of continuous variables was assessed using visual inspection of histograms and the Shapiro–Wilk test. As most continuous variables deviated from normality, non-parametric methods were applied. Group differences in continuous variables were examined using the Kruskal–Wallis test. When a significant omnibus effect was detected, pairwise post hoc comparisons were performed using Mann–Whitney U tests with Bonferroni-adjusted significance thresholds.

Categorical variables were compared using Pearson’s chi-square test. Fisher’s exact test was applied when expected cell counts were < 5. For analyses restricted to the encopresis groups (ENC vs. ENC + ADHD), group comparisons were conducted using chi-square tests for categorical variables and Mann–Whitney U tests for continuous variables.

To examine the independent and interactive associations of ENC and ADHD with emotional, behavioral, eating-related, and executive-functioning outcomes, a series of 2 × 2 factorial linear regression models was constructed in Stata. Here, the term factorial refers to the analytic framework rather than to an experimental study design. ENC (present vs. absent) and ADHD (present vs. absent) were entered as fixed factors together with their interaction term (ENC × ADHD). ENC and ADHD status were modeled as two observed clinical factors, rather than experimentally manipulated conditions. To address reviewer concerns regarding potential overadjustment by global clinical severity, the factorial regression analyses were conducted in two stages. In Panel A, models were adjusted for age (in months), sex, and body mass index z-score (BMI z-score). In Panel B, the same models were additionally adjusted for clinician-rated global severity, as assessed by the CGI-S.

Heteroscedasticity-consistent HC3 robust standard errors were used in all regression models. For each outcome, the significance of main and interaction effects was evaluated using Wald tests. Adjusted marginal means with standard errors were estimated for each diagnostic combination (No ENC/No ADHD, No ENC/ADHD, ENC/No ADHD, ENC/ADHD) and are presented in the main regression table. In models showing nominally significant interaction effects, post-estimation marginal analyses were used to inspect the pattern of adjusted group differences. To evaluate the robustness of the interaction findings, supplementary analyses were conducted for all outcomes. These included permutation-based *p* values, bootstrap resampling estimates summarized as the bootstrap median and 95% confidence interval, and model fit indices (R^2^ and adjusted R^2^). Because a large number of factorial models were tested, false discovery rate (FDR) correction was applied to the interaction *p* values based on both HC3-derived and permutation-derived estimates. These robustness analyses are reported in Supplementary Tables S1 and S2.

All statistical tests were two-tailed. A *p* value < 0.05 was considered statistically significant for primary analyses. Where multiple post hoc comparisons were performed, appropriate corrections were applied as described above. Results are reported with corresponding test statistics and exact *p* values. Because the interaction analyses involved multiple comparisons across a broad set of outcomes, interaction effects were interpreted primarily in light of the supplementary robustness analyses, particularly FDR-corrected results.

## Results

### Sociodemographic and Clinical Characteristics

The sample consisted of four groups: typically developing controls (HC; *n* = 31), ADHD-only (*n* = 60), ENC-only (*n* = 31), and ENC + ADHD (*n* = 26) (Table 1). Sex distribution differed significantly across groups (*χ*^2^ = 10.38, *p* = 0.016), with a marked male predominance in the ENC + ADHD group (92.3%) and a higher proportion of females in the ENC-only group (45.2%). Groups did not differ in age, height, weight, or BMI z-scores (all *p* > 0.05), indicating good anthropometric comparability. Family medical and psychiatric history variables were also comparable across groups.

**Table 1:** Sociodemographic and clinical characteristics of the study groups

		HC (*n* = 31)	ADHD (*n* = 60)	ENC (*n* = 31)	ENC + ADHD (*n* = 26)	χ^2^ or H	*p*	*Effect size*
Sex	Female	12 (38.7)	18 (30.0)	14 (45.2)	2 (7.7)	10.38	**0.016**	*V* = 0.265
	Male	19 (61.3)	42 (70.0)	17 (54.8)	24 (92.3)			
Age (months)		89.2 ± 17.9/ 86 (61–119)	93.8 ± 18.2/ 96 (62–120)	93.0 ± 20.0/ 89 (63–120)	91.2 ± 18.9/ 89 (60–120)	1.47	0.689	*ε*^2^ = 0.000
Height (cm)		124.6 ± 10.6/ 124 (109–147)	127.2 ± 12.8/ 126 (103–160)	128.8 ± 14.5/ 130 (107–157)	125.8 ± 13.0/ 124 (106–147)	1.76	0.625	*ε*^2^ = 0.000
Weight (kg)		27.0 ± 8.5/ 26 (16–43)	28.9 ± 9.3/ 27.5 (17–61)	29.1 ± 9.9/ 27 (16–61)	29.2 ± 9.9/ 25 (19–49)	1.06	0.786	*ε*^2^ = 0.000
BMI z-score		0.18 ± 1.57/ − 0.15 (− 2.69–3.05)	0.39 ± 1.24/ 0.52 (− 3.16–2.84)	− 0.07 ± 2.25/ 0.44 (− 6.52–2.46)	0.63 ± 1.47/ 0.92 (− 2.29–2.33)	1.75	0.626	*ε*^2^ = 0.000
Family history of medical illness		4 (12.9)	11 (18.3)	6 (19.4)	4 (15.4)	0.62	0.893	*V* = 0.065
Sibling psychiatric disorder		6 (19.4)	6 (10.0)	6 (19.4)	4 (15.4)	2.12	0.549	*V* = 0.120
Family history of psychiatric disorder		10 (32.3)	13 (21.7)	7 (22.6)	4 (15.4)	2.42	0.490	*V* = 0.128
CGI–Severity		1.0 ± 0.0/ 1 (1–1)	4.7 ± 0.7/ 5 (3–7)	5.2 ± 0.8/ 5 (4–7)	5.7 ± 1.1/ 6 (4–7)	89.84	** < 0.001**	*ε*^2^ = 0.603
Age of ADHD onset (months)		—	56.5 ± 17.0/ 60.0 (12–84)	—	58.8 ± 18.7/ 60.0 (12–90)	U/Z = 725.0/−0.54	0.590	*r* = 0.058
ADHD type		—	60 (100.0)	—	26 (100.0)	8.69	**0.013**	*V* = 0.318
ADHD-I		—	16 (26.7)	—	0 (0.0)			
ADHD-HI		—	12 (20.0)	—	6 (23.1)			
ADHD-C		—	32 (53.3)	—	**20 (76.9)**			
Language disorder		0 (0.0)	4 (6.7)	4 (12.9)	0 (0.0)	6.85	0.077	*V* = 0.215
EN		0 (0.0)	9 (15.0)	**14 (45.2)**	2 (7.7)	25.67	** < 0.001**	*V* = 0.416
ODD		0 (0.0)	5 (8.3)	2 (6.5)	**6 (23.1)**	9.84	**0.020**	*V* = 0.258
ARFID		0 (0.0)	1 (1.7)	0 (0.0)	0 (0.0)	1.48	0.688	*V* = 0.100
Any stressor in the past month		2 (6.5)	3 (5.0)	9 (29.0)	2 (7.7)	13.65	**0.003**	*V* = 0.304
Parental divorce/separation in the past month		0 (0.0)	0 (0.0)	2 (6.5)	0 (0.0)	7.65	0.054	*V* = 0.227
Bereavement or serious illness in the past month		1 (3.2)	0 (0.0)	0 (0.0)	0 (0.0)	3.80	0.284	*V* = 0.160
Peer bullying in the past month		0 (0.0)	0 (0.0)	3 (9.7)	2 (7.7)	8.43	**0.038**	*V* = 0.239
Change of home/school/city/teacher in the past month		0 (0.0)	1 (1.7)	2 (6.5)	2 (7.7)	4.00	0.261	*V* = 0.164
Birth of a sibling in the past month		0 (0.0)	1 (1.7)	2 (6.5)	0 (0.0)	4.27	0.233	*V* = 0.170

Values are presented as n (%) for categorical variables and as mean ± standard deviation/median (minimum–maximum) for continuous variables. Group comparisons were performed using chi-square (χ^2^) tests for categorical variables and Kruskal–Wallis H tests for non-normally distributed continuous variables. For pairwise comparisons, Mann–Whitney U tests were used where applicable. Post hoc group differences for CGI–Severity followed the pattern HC < ADHD < ENC = ENC + ADHD. *HC* healthy controls; *ADHD* attention-deficit/hyperactivity disorder; *ENC* encopresis; *ENC + ADHD* comorbid encopresis and ADHD; *BMI* body mass index; *CGI–Severity* Clinical Global Impression–Severity scale; *ADHD-I* predominantly inattentive presentation; *ADHD-HI* predominantly hyperactive–impulsive presentation; *ADHD-C* combined presentation; *EN* enuresis; *ODD* oppositional defiant disorder; *ARFID* avoidant/restrictive food intake disorder

Clinical severity differed significantly (*H* = 89.84, *p* < 0.001), following a graded pattern (HC < ADHD-only < ENC-only = ENC + ADHD), with the highest CGI-S scores observed in the ENC + ADHD group. Among children with ADHD, age at onset did not differ between ADHD-only and ENC + ADHD groups (*p* = 0.590), whereas ADHD subtype distribution differed significantly (*χ*^2^ = 8.69, *p* = 0.013), with the combined subtype predominating in the ENC + ADHD group. ENC and ODD prevalence differed significantly across groups (both *p* < 0.05), with the highest ODD rates in the ENC + ADHD group. Language disorder showed a trend-level difference, while ARFID was rare and did not differ between groups.

Exposure to recent psychosocial stressors differed significantly (*χ*^2^ = 13.65, *p* = 0.003), being most frequent in the ENC group. Peer bullying also varied across groups (*χ*^2^ = 8.43, *p* = 0.038), occurring mainly in the ENC and ENC + ADHD groups. Other stressors did not show significant group differences.

### Toileting-Related Behaviors and Constipation Severity

Significant group differences were observed in toileting behaviors and constipation-related variables (Table 2). Toilet training completion differed markedly (*χ*^2^ = 34.29, *p* < 0.001), with near-universal completion in HC and ADHD-only, but substantially lower rates in ENC-only (67.7%) and ENC + ADHD (76.9%). Stool consistency also varied significantly (*χ*^2^ = 44.02, *p* < 0.001); Type 4 stools predominated in HC and ADHD-only, whereas harder stool types were more common in ENC, particularly ENC + ADHD.

**Table 2:** Toileting-related characteristics, bowel habits, and constipation severity across diagnostic groups

		HC (*n* = 31)	ADHD (*n* = 60)	ENC (*n* = 31)	ENC + ADHD (*n* = 26)	χ^2^	*p*	*Effect size*
Toilet training status	Completed	31 (100.0)	59 (98.3)	21 (67.7)	20 (76.9)	34.29	** < 0.001**	*V* = 0.340
	Partially completed	0 (0.0)	1 (1.7)	6 (19.4)	6 (23.1)			
	Not completed	0 (0.0)	0 (0.0)	4 (12.9)	0 (0.0)			
Bristol stool form scale	Type 1	0 (0.0)	0 (0.0)	6 (19.4)	2 (7.7)	44.02	** < 0.001**	*V* = 0.315
	Type 2	0 (0.0)	3 (5.0)	6 (19.4)	2 (7.7)			
	Type 3	7 (22.6)	18 (30.0)	10 (32.3)	10 (38.5)			
	Type 4	23 (74.2)	36 (60.0)	7 (22.6)	8 (30.8)			
	Type 5	1 (3.2)	2 (3.3)	0 (0.0)	2 (7.7)			
	Type 6	0 (0.0)	1 (1.7)	2 (6.5)	2 (7.7)			
≥ 1 fecal incontinence episode per month (past 3 months)		0 (0.0)	0 (0.0)	31 (100.0)	26 (100.0)	—	—	—
≥ 1 fecal incontinence episode per week (past 1 month)		0 (0.0)	0 (0.0)	31 (100.0)	26 (100.0)	—	—	—
< 2 bowel movements per week		0 (0.0)	0 (0.0)	6 (19.4)	16 (61.5)	61.14	** < 0.001**	*V* = 0.643
Retentive posturing/stool withholding		0 (0.0)	0 (0.0)	16 (51.6)	7 (26.9)	50.04	** < 0.001**	*V* = 0.581
Painful or hard bowel movements		0 (0.0)	0 (0.0)	12 (38.7)	4 (15.4)	36.62	** < 0.001**	*V* = 0.497
Large-diameter stools that may obstruct the toilet		0 (0.0)	0 (0.0)	0 (0.0)	4 (15.4)	19.29	**0.001**	*V* = 0.361
Fecal mass in rectum		0 (0.0)	0 (0.0)	4 (12.9)	4 (15.4)	13.67	**0.003**	*V* = 0.304
Encopresis subtype	Retentive	—	—	16 (51.6)	**22 (84.6)**	6.93	**0.008**	*V* = 0.349
	Non-retentive	—	—	15 (48.4)	4 (15.4)			
Onset type	Secondary	—	—	15 (48.4)	10 (38.5)	0.57	0.452	*V* = 0.100
	Primary	—	—	16 (51.6)	16 (61.5)			
Dry period duration, if achieved (months)		—	—	21.2 ± 14.2/ 18.0 (12–70)	18.4 ± 7.7/ 18.0 (10–30)	U/Z = 69.0/−0.34	0.737	—
Age at first toilet training attempt (months)		26.7 ± 5.1/ 24.0 (18–36)	28.0 ± 6.9/ 24.0 (18–48)	28.2 ± 7.2/ 24.0 (20–48)	30.9 ± 7.1/ 36.0 (18–42)	5.44	0.142	*ε*^2^ = 0.017
Duration of toilet training (days)		29.0 ± 35.3/ 14.0 (2–150)	35.3 ± 115.1/ 14.0 (3–900)	26.1 ± 15.4/ 24.0 (7–60)	26.5 ± 18.1/ 21.0 (7–60)	6.53	0.088	*ε*^2^ = 0.025
Number of failed attempts (count)		0.1 ± 0.3/ 0.0 (0–1)	0.9 ± 6.5/ 0.0 (0–50)	8.4 ± 16.9/ 0.0 (0–60)	1.7 ± 3.7/ 0.0 (0–10)	20.00	** < 0.001**	*ε*^2^ = 0.118
Weekly bowel movements (count/week)		6.8 ± 1.4/ 7.0 (3–12)	7.0 ± 1.3/ 7.0 (3–14)	9.8 ± 12.3/ 6.0 (2–50)	6.5 ± 3.2/ 7.0 (1–14)	6.73	0.081	*ε*^2^ = 0.026
Daily water intake (glasses/day)		7.3 ± 1.8/ 7.5 (4–10)	8.2 ± 2.1/ 8.0 (4–15)	7.4 ± 3.7/ 5.0 (4–20)	7.5 ± 2.5/ 7.0 (4–10)	6.66	0.084	*ε*^2^ = 0.025
Days with moderate-to-vigorous physical activity (days/week)		5.0 ± 1.9/ 5.0 (1–7)	6.3 ± 1.3/ 7.0 (3–7)	5.6 ± 1.9/ 7.0 (1–7)	5.9 ± 1.6/ 7.0 (3–7)	12.00	**0.007**	*ε*^2^ = 0.062
Daily duration of moderate-to-vigorous activity (minutes/day)		86.1 ± 34.4/ 60.0 (30–180)	121.5 ± 52.8/ 120.0 (60–300)	108.4 ± 62.8/ 120.0 (30–300)	83.1 ± 27.2/ 60.0 (60–120)	16.14 (3)	**0.001**	*ε*^2^ = 0.091
Screen time (hours/day)		1.9 ± 0.9/ 2.0 (1–4)	2.3 ± 0.9/ 2.0 (1–5)	2.7 ± 1.5/ 2.0 (1–6)	2.3 ± 1.0/ 2.0 (1–4)	6.12 (3)	0.106	*ε*^2^ = 0.022

Values are presented as n (%) for categorical variables and as mean ± standard deviation/median (minimum–maximum) for continuous variables. Group comparisons were conducted using chi-square (χ^2^) tests for categorical variables and Kruskal–Wallis H tests for continuous variables that did not meet normality assumptions. When appropriate, Mann–Whitney U tests were used for post hoc pairwise comparisons. Post hoc comparisons for the number of failed toilet training attempts indicated the pattern HC = ADHD < ENC = ENC + ADHD. For days of moderate-to-vigorous physical activity per week, post hoc analyses showed higher values in the ADHD group compared with HC, while ENC and ENC + ADHD did not differ significantly from either group. For daily duration of moderate-to-vigorous physical activity, the ADHD and ENC groups showed longer activity durations compared with HC and ENC + ADHD. *HC* healthy controls; *ADHD* attention-deficit/hyperactivity disorder; *ENC* encopresis; *ENC + ADHD* comorbid encopresis and ADHD

By definition, fecal incontinence was present in all ENC and ENC + ADHD participants and absent in HC and ADHD-only. Constipation severity indicators—including infrequent bowel movements, stool withholding, painful defecation, large-diameter stools, and fecal mass—were significantly more prevalent in ENC-only and ENC + ADHD groups (all *p* ≤ 0.003). Retentive encopresis was more frequent in the ENC + ADHD group than in ENC-only (*χ*^2^ = 6.93, *p* = 0.008). ENC onset type and dry period duration did not differ between groups.

The number of failed toilet training attempts differed significantly (*H* = 20.00, *p* < 0.001), with higher counts in ENC and ENC + ADHD compared with HC and ADHD-only. Age at first toilet training attempt and training duration were comparable across groups. Physical activity frequency (*H* = 12.00, *p* = 0.007) and daily duration (*H* = 16.14, *p* = 0.001) differed across groups, with higher activity levels in ADHD-only and ENC-only groups. No group differences were observed for bowel movement frequency, water intake, or screen time.

### Internalizing and Externalizing Symptoms, Eating Behaviors, and Executive Functioning

Most SDQ subscales differed significantly across groups, except for prosocial behavior (Table 3). Emotional symptoms, internalizing problems, and total difficulties followed a graded increase from HC and ADHD-only to ENC-only and ENC + ADHD (all *p* < 0.001). Peer problems and conduct problems were higher in ENC groups, while hyperactivity was most pronounced in ADHD-only, followed by ENC + ADHD. Externalizing problems were elevated in all clinical groups relative to HC.

**Table 3:** Internalizing and externalizing symptoms, and eating behaviors across diagnostic groups

	HC (*n* = 31)	ADHD (*n* = 60)	ENC (*n* = 31)	ENC + ADHD (*n* = 26)	*H*	*p*	Post hoc	Effect size
**SDQ**								
Emotional Symptoms	3.0 ± 2.6/ 3 (0–10)	3.1 ± 2.2/ 3 (0–9)	4.9 ± 2.8/ 5 (0–9)	6.2 ± 1.9/ 6 (3–9)	32.13	** < 0.001**	**0 = 1 < 2 = 3**	*ε*^2^ = 0.202
Peer Problems	2.9 ± 2.2/ 2 (0–7)	3.3 ± 1.9/ 3.5 (0–8)	4.3 ± 2.4/ 4 (0–9)	5.0 ± 1.8/ 5 (3–8)	16.57	**0.001**	**0 < 2 = 3, 0 = 1, 1 = 2, 1 < 3**	*ε*^2^ = 0.094
Conduct Problems	1.6 ± 1.3/ 2 (0–4)	3.4 ± 2.2/ 3 (0–9)	4.4 ± 3.3/ 4 (0–9)	5.9 ± 2.5/ 5 (2–9)	36.79	** < 0.001**	**0 < 1 = 2 < 3**	*ε*^2^ = 0.235
Hyperactivity	3.3 ± 2.2/ 3 (0–9)	6.6 ± 2.3/ 7 (2–16)	3.9 ± 1.9/ 4 (0–6)	5.5 ± 1.4/ 6 (3–8)	48.56	** < 0.001**	**1 > 3 > 2 = 0**	*ε*^2^ = 0.316
Internalizing Problems	5.8 ± 3.9/ 6 (0–12)	6.5 ± 3.2/ 6 (1–14)	9.2 ± 4.7/ 9 (3–17)	11.2 ± 2.9/ 11 (7–15)	31.85	** < 0.001**	**0 = 1 < 2 = 3**	*ε*^2^ = 0.200
Externalizing Problems	4.9 ± 2.9/ 5 (0–11)	10.0 ± 3.6/ 10 (2–21)	8.3 ± 4.7/ 9 (2–15)	11.5 ± 2.7/ 11 (5–15)	43.63	** < 0.001**	**0 < 1 = 2 < 3**	*ε*^2^ = 0.282
Prosocial Behavior	8.1 ± 1.8/ 9 (4–10)	7.6 ± 1.9/ 8 (1–10)	7.5 ± 1.9/ 8 (4–10)	7.1 ± 1.9/ 7 (4–10)	4.24	0.236	—	*ε*^2^ = 0.009
Total Difficulties	10.8 ± 5.9/ 10 (0–23)	16.5 ± 5.8/ 16 (7–33)	17.5 ± 8.9/ 17 (5–30)	22.6 ± 5.3/ 23 (13–30)	36.16	** < 0.001**	**0 < 1 = 2 < 3**	*ε*^2^ = 0.230
**CPRS-27**								
Oppositional	4.0 ± 3.4/ 3 (0–14)	9.5 ± 4.3/ 9 (1–18)	8.5 ± 4.6/ 7 (3–17)	10.9 ± 3.5/ 11 (4–17)	42.73	** < 0.001**	**0 < 1 = 2, 0 < 2 < 3, 1 = 3**	*ε*^2^ = 0.276
Inattention	2.7 ± 2.8/ 2 (0–12)	11.9 ± 3.5/ 11.5 (3–18)	5.5 ± 5.5/ 3 (0–18)	11.5 ± 2.7/ 12 (6–15)	72.52	** < 0.001**	**0 < 2 < 1 = 3**	*ε*^2^ = 0.483
Hyperactivity	3.1 ± 3.3/ 2 (0–10)	9.6 ± 4.1/ 9 (1–17)	6.1 ± 5.4/ 4 (0–16)	11.2 ± 3.9/ 12 (3–17)	49.72	** < 0.001**	**0 < 2 < 1 = 3**	*ε*^2^ = 0.324
ADHD Index	6.7 ± 5.3/ 5 (0–19)	22.3 ± 5.8/ 22.5 (10–33)	13.6 ± 9.4/ 9 (3–34)	22.9 ± 4.9/ 22 (13–30)	71.72	** < 0.001**	**0 < 2 < 1 = 3**	*ε*^2^ = 0.477
Total Score	15.3 ± 11.9/13 (0–44)	47.6 ± 12.8/ 45.5 (17–72)	31.2 ± 20.3/ 25 (11–74)	51.1 ± 11.6/ 51 (24–67)	68.68	** < 0.001**	**0 < 2 < 1 = 3**	*ε*^2^ = 0.456
**NIAS**								
Picky Eating	9.0 ± 4.6/ 8 (3–18)	11.0 ± 4.7/ 11.5 (3–18)	9.8 ± 3.9/ 9 (3–18)	11.4 ± 3.0/ 12 (7–18)	7.07	0.070	—	*ε*^2^ = 0.028
Appetite	8.3 ± 4.3/ 6 (3–18)	9.1 ± 4.5/ 8.5 (3–18)	7.9 ± 4.2/ 6 (3–16)	8.2 ± 3.4/ 7 (3–16)	3.32	0.344	—	*ε*^2^ = 0.002
Fear	5.5 ± 3.0/ 5 (3–13)	5.3 ± 3.2/ 3.5 (3–18)	3.9 ± 1.8/ 3 (3–10)	6.9 ± 2.4/ 7 (3–11)	25.00	** < 0.001**	**2 < 0 = 1 < 3**	*ε*^2^ = 0.153
Total Score	22.8 ± 9.2/ 20 (9–41)	25.3 ± 9.2/ 25 (9–46)	21.6 ± 6.6/ 22 (9–37)	26.5 ± 5.0/ 28 (17–35)	9.71	0.021	—	*ε*^2^ = 0.047

Values are presented as mean ± standard deviation/median (minimum–maximum). Group differences were examined using Kruskal–Wallis H tests due to non-normal distribution of outcome variables. When overall group differences were significant, pairwise post hoc comparisons were performed using Mann–Whitney U tests with Bonferroni adjustment. Post hoc results are presented using group codes, where 0 = HC, 1 = ADHD, 2 = ENC, and 3 = ENC + ADHD. *HC* healthy controls; *ADHD* attention-deficit/hyperactivity disorder; *ENC* encopresis; *ENC + ADHD* comorbid encopresis and ADHD; *SDQ* Strengths and Difficulties Questionnaire; *CPRS-27* Conners’ Parent Rating Scale–27; *NIAS* Nine Item Avoidant/Restrictive Food Intake Disorder Screen

All CPRS-R:S subscales showed significant group differences (all *p* < 0.001). ADHD-only and ENC + ADHD groups exhibited the highest inattention, hyperactivity, ADHD Index, and total scores, with intermediate scores in the ENC-only group.

NIAS analyses revealed no group differences for picky eating or appetite. In contrast, fear-related eating differed significantly (*H* = 25.00, *p* < 0.001), peaking in the ENC + ADHD group. The NIAS total score also differed across groups (*p* = 0.021), with higher scores in ADHD- only and ENC + ADHD.

All BRIEF subscales, indices, and composite scores differed significantly between groups (Table 4). Executive dysfunction showed a consistent gradient, with the greatest impairment in ENC + ADHD, followed by ADHD-only, ENC-only, and HC. This pattern was evident across inhibition, shifting, planning, working memory, monitoring, BRI, MI, GEC, and total scores. Validity scales (Negativity and Inconsistency) were also higher in ADHD-only and ENC + ADHD groups.

**Table 4:** Executive functioning profiles across diagnostic groups as assessed by the BRIEF

	HC (*n* = 31)	ADHD (*n* = 60)	ENC (*n* = 31)	ENC + ADHD (*n* = 26)	*H*	*p*	Post hoc	Effect size
Emotional Control	16.9 ± 4.5/ 17 (10–29)	20.9 ± 4.4/ 19.5 (15–30)	19.6 ± 5.1/ 18 (13–28)	23.2 ± 4.8/ 24 (16–30)	21.47	** < 0.001**	**0 = 2 < 3, 0 < 1, 1 = 2, 1 = 3**	*ε*^2^ = 0.128
Shift	16.6 ± 4.1/ 16 (11–27)	21.9 ± 3.9/ 21 (15–32)	20.4 ± 3.7/ 20 (13–28)	23.4 ± 2.8/ 23 (19–29)	38.69	** < 0.001**	**0 < 2 < 3, 0 < 1 = 2, 0 < 2 = 3**	*ε*^2^ = 0.248
Inhibit	20.5 ± 5.0/ 20 (15–30)	31.3 ± 6.2/ 31 (18–43)	24.9 ± 6.0/ 23 (17–35)	31.4 ± 5.7/ 34 (23–41)	55.66	** < 0.001**	**0 < 2 < 1 = 3**	*ε*^2^ = 0.366
Plan/Organize	21.5 ± 5.6/ 20 (15–35)	31.7 ± 4.9/ 32 (22–45)	26.1 ± 5.5/ 25 (18–37)	33.2 ± 3.5/ 33 (29–40)	59.94	** < 0.001**	**0 < 2 < 1 = 3**	*ε*^2^ = 0.395
Working Memory	17.2 ± 4.9/ 16 (11–31)	24.0 ± 3.1/ 24 (17–32)	19.1 ± 4.1/ 19 (12–25)	23.9 ± 2.7/ 24 (17–29)	55.82	** < 0.001**	**0 < 2 < 1 = 3**	*ε*^2^ = 0.367
Initiate	11.7 ± 2.9/ 11 (8–20)	16.2 ± 2.7/ 16 (11–22)	14.2 ± 3.5/ 14 (9–20)	17.3 ± 2.0/ 17 (14–21)	46.88	** < 0.001**	**0 < 2 < 1 < 3**	*ε*^2^ = 0.305
Organization of Materials	11.1 ± 3.2/ 10 (8–22)	15.9 ± 3.5/ 16 (9–24)	13.9 ± 4.2/ 14 (9–23)	16.2 ± 3.9/ 16 (10–23)	39.41	** < 0.001**	**0 < 2 < 1 = 3**	*ε*^2^ = 0.253
Monitor	12.5 ± 3.6/ 12 (8–20)	17.9 ± 2.8/ 18 (12–24)	15.5 ± 3.0/ 14 (10–21)	18.7 ± 2.1/ 18 (16–23)	46.78	** < 0.001**	**0 < 2 < 1 = 3**	*ε*^2^ = 0.304
Behavioral Regulation Index	54.0 ± 12.5/ 53 (36–86)	74.1 ± 12.4/ 72.5 (55–101)	64.8 ± 12.3/ 62 (45–88)	77.9 ± 11.5/ 78 (62–100)	45.90	** < 0.001**	**0 < 2 < 1 = 3**	*ε*^2^ = 0.298
Metacognition Index	73.9 ± 17.1/ 70 (51–127)	105.7 ± 12.7/ 105.5 (78–139)	88.8 ± 17.9/ 84 (61–114)	109.2 ± 10.0/ 109 (90–130)	59.59	** < 0.001**	**0 < 2 < 1 = 3**	*ε*^2^ = 0.393
Global Executive Composite	127.9 ± 28.5/ 120 (87–213)	179.8 ± 22.7/ 178 (137–240)	153.6 ± 28.9/ 145 (106–201)	187.2 ± 19.8/ 184 (168–230)	59.32	** < 0.001**	**0 < 2 < 1 = 3**	*ε*^2^ = 0.391
Negativity Scale	0.8 ± 1.6/ 0 (0–6)	2.2 ± 2.0/ 2 (0–7)	1.8 ± 1.8/ 1 (0–6)	3.3 ± 2.0/ 3 (1–7)	30.92	** < 0.001**	**0 < 1 = 2 < 3**	*ε*^2^ = 0.194
Inconsistency Scale	4.1 ± 2.1/ 4 (0–7)	5.8 ± 2.2/ 6 (1–10)	5.4 ± 2.0/ 5 (2–9)	6.2 ± 2.4/ 5 (4–11)	11.43	**0.010**	**0 < 1 = 3, 1 = 2 = 3, 0 = 2**	*ε*^2^ = 0.059
Total Score	110.2 ± 24.2/ 105 (74–180)	153.2 ± 19.4/ 152 (117–201)	132.2 ± 23.9/ 129 (93–169)	158.6 ± 17.8/ 157 (137–195)	57.24	** < 0.001**	**0 < 2 < 1 = 3**	*ε*^2^ = 0.377

Values are presented as mean ± standard deviation/median (minimum–maximum). Group differences were examined using Kruskal–Wallis H tests due to non-normal distributions of BRIEF scores. When overall group differences were significant, pairwise post hoc comparisons were conducted using Mann–Whitney U tests with Bonferroni correction. Post hoc results are reported using group codes, where 0 = HC, 1 = ADHD, 2 = ENC, and 3 = ENC + ADHD. *HC* healthy controls; *ADHD* attention-deficit/hyperactivity disorder; *ENC* encopresis; *ENC + ADHD* comorbid encopresis and ADHD. *BRIEF* Behavior Rating Inventory of Executive Function; *EC* Emotional Control; *BRI* Behavioral Regulation Index; *MI* Metacognition Index; *GEC* Global Executive Composite

### Factorial Regression Analyses of ENC and ADHD Effects

**Fig. 1:**
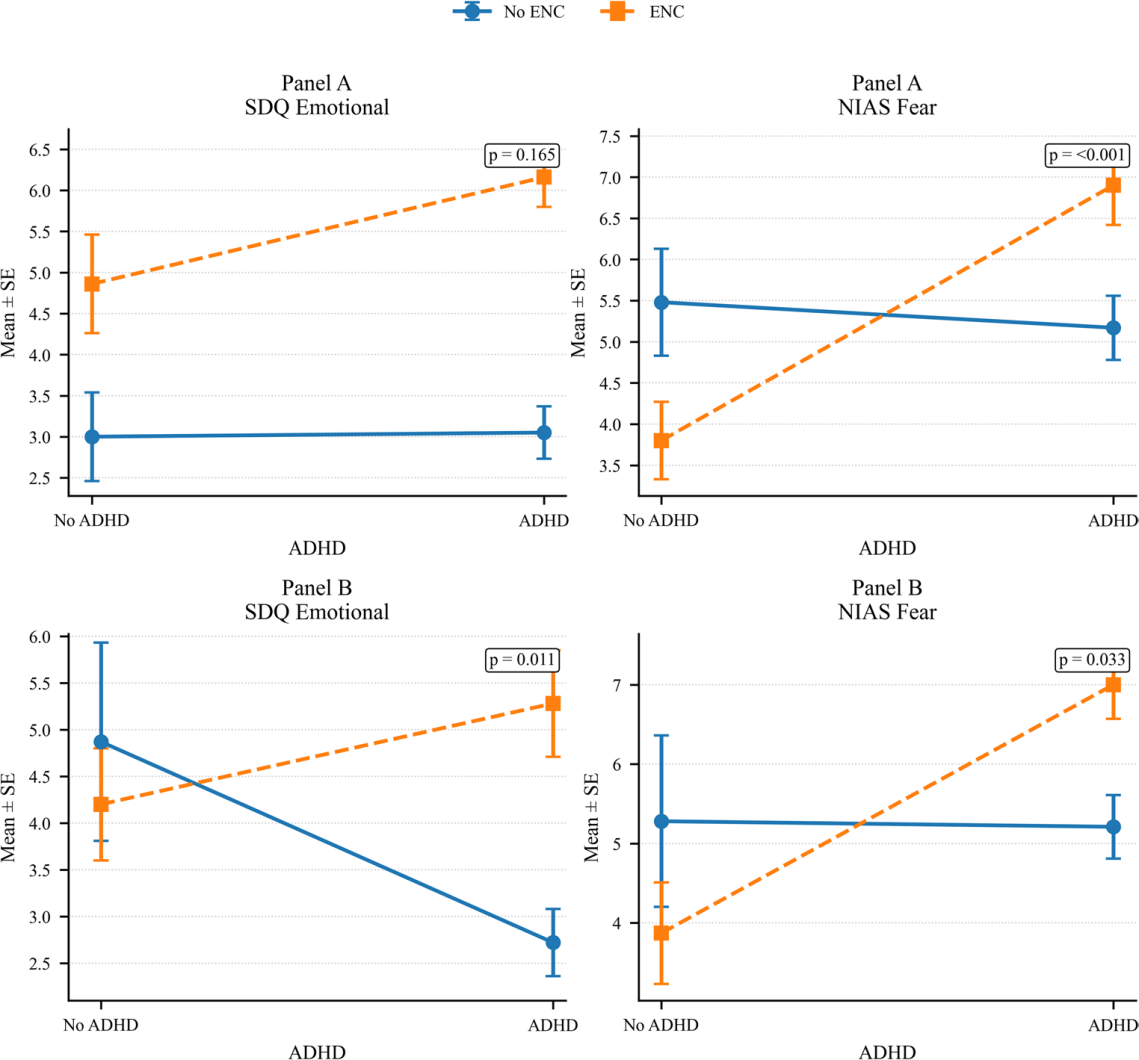
Main and interaction effects of encopresis (ENC) and attention-deficit/hyperactivity disorder (ADHD) on SDQ emotional symptoms and NIAS fear scores. *Note.* Results are based on 2 × 2 factorial linear regression models examining the main effects of encopresis (ENC: present vs. absent), attention-deficit/hyperactivity disorder (ADHD: present vs. absent), and their interaction (ENC × ADHD). Panel A presents models adjusted for age (months), sex, and BMI z-score. Panel B additionally includes clinician-rated global severity (CGI-S) as a covariate. Values are presented as estimated marginal means with standard errors (SE) for each group

To assess the independent and interactive associations of ENC and ADHD, 2 × 2 factorial regression models were conducted in two stages: Panel A models were adjusted for age, sex, and BMI z-score, whereas Panel B models additionally adjusted for CGI-S (Table 5). Adjusted marginal means for the outcomes showing the most relevant interaction patterns are presented in Fig. 1. Full model results, including HC3-robust inference, permutation-based *p* values, bootstrap estimates, and FDR-corrected interaction *p* values, are presented in Supplementary Tables S1 and S2.

**Table 5:** Main and ınteraction effects of ENC and ADHD on emotional, behavioral, eating, and executive functioning outcomes (sex, age, BMI z-score adjusted)

Outcome	No ENC/No ADHD *Mean (SE)*	No ENC/ADHD *Mean (SE)*	ENC/No ADHD *Mean (SE)*	ENC/ADHD *Mean (SE)*	ENC β	ENC p	ADHD β	ADHD p	Interaction β	Interaction p
**Panel A. Models Adjusted For Age (Months), Sex, And BMI Z-Score**										
SDQ Emotional Symptoms	3.00 (0.54)	3.05 (0.32)	4.86 (0.60)	6.16 (0.36)	1.851	**0.012**	0.041	0.944	1.259	0.165
SDQ Peer Problems	3.02 (0.42)	3.37 (0.28)	4.37 (0.46)	5.07 (0.39)	1.354	**0.018**	0.355	0.450	0.348	0.636
SDQ Conduct Problems	1.74 (0.31)	3.37 (0.31)	4.36 (0.64)	5.95 (0.48)	2.616	** < 0.001**	1.622	** < 0.001**	−0.037	0.966
SDQ Hyperactivity	3.44 (0.43)	6.77 (0.28)	4.12 (0.46)	5.58 (0.29)	0.675	0.217	3.331	** < 0.001**	−1.863	**0.006**
SDQ Internalizing	6.02 (0.79)	6.42 (0.47)	9.23 (0.94)	11.23 (0.57)	3.205	**0.005**	0.395	0.645	1.606	0.245
SDQ Externalizing	5.19 (0.64)	10.14 (0.47)	8.48 (0.99)	11.53 (0.52)	3.291	**0.002**	4.953	** < 0.001**	−1.901	0.138
SDQ Prosocial	8.00 (0.37)	7.56 (0.28)	7.35 (0.41)	7.10 (0.39)	−0.647	0.192	−0.442	0.292	0.191	0.784
SDQ Total Difficulties	11.21 (1.27)	16.55 (0.82)	17.70 (1.80)	22.76 (1.00)	6.496	**0.001**	5.348	** < 0.001**	−0.294	0.904
CPRS-Oppositional	4.19 (0.85)	9.63 (0.61)	8.58 (0.87)	11.13 (0.62)	4.393	** < 0.001**	5.445	** < 0.001**	−2.895	**0.026**
CPRS-Inattention	2.49 (0.57)	11.64 (0.55)	5.17 (1.06)	11.38 (0.51)	2.683	**0.014**	9.148	** < 0.001**	−2.943	**0.028**
CPRS-Hyperactivity	3.28 (0.69)	9.69 (0.60)	6.28 (1.06)	11.23 (0.79)	3.001	**0.009**	6.407	** < 0.001**	−1.462	0.332
CPRS-ADHD Index	6.59 (1.14)	22.05 (0.91)	13.48 (1.80)	22.73 (0.97)	6.893	** < 0.001**	15.461	** < 0.001**	−6.216	0.008
CPRS-Total Score	15.46 (2.64)	47.38 (1.97)	31.13 (4.01)	51.11 (2.26)	15.674	** < 0.001**	31.923	** < 0.001**	−11.945	0.021
NIAS Picky Eating	9.11 (0.99)	11.12 (0.66)	9.85 (0.82)	11.57 (0.62)	0.736	0.502	2.013	0.063	−0.295	0.833
NIAS Appetite	7.54 (0.89)	8.62 (0.58)	6.76 (0.79)	8.37 (0.65)	−0.775	0.478	1.087	0.274	0.524	0.703
NIAS Fear	5.48 (0.65)	5.17 (0.39)	3.80 (0.47)	6.90 (0.48)	−1.684	**0.009**	−0.312	0.665	3.412	** < 0.001**
NIAS Total	22.13 (2.02)	24.92 (1.23)	20.41 (1.44)	26.84 (0.92)	−1.723	0.418	2.789	0.201	3.642	0.155
BRIEF Emotional Control	16.95 (0.83)	20.95 (0.63)	19.70 (1.05)	23.16 (1.00)	2.745	**0.032**	3.998	** < 0.001**	−0.531	0.762
BRIEF Shift	16.82 (0.80)	22.07 (0.54)	20.47 (0.77)	23.57 (0.59)	3.647	** < 0.001**	5.245	** < 0.001**	−2.142	0.098
BRIEF Inhibit	20.58 (1.05)	31.39 (0.86)	24.77 (1.22)	31.57 (1.23)	4.198	**0.004**	10.811	** < 0.001**	−4.017	0.057
BRIEF Plan	21.73 (1.13)	31.79 (0.71)	26.20 (1.14)	33.25 (0.77)	4.471	**0.002**	10.054	** < 0.001**	−3.013	0.096
BRIEF Working Memory	17.29 (0.91)	24.01 (0.45)	19.26 (0.90)	23.88 (0.57)	1.970	0.105	6.724	** < 0.001**	−2.105	0.147
BRIEF Initiate	11.70 (0.52)	16.14 (0.37)	14.17 (0.74)	17.25 (0.40)	2.466	**0.004**	4.434	** < 0.001**	−1.349	0.194
BRIEF OFM	10.76 (0.61)	15.65 (0.50)	13.39 (0.88)	16.21 (0.77)	2.623	**0.008**	4.885	** < 0.001**	−2.061	0.142
BRIEF Monitor	12.81 (0.74)	18.09 (0.40)	15.82 (0.66)	18.78 (0.45)	3.014	** < 0.001**	5.281	** < 0.001**	−2.320	**0.028**
BRIEF BRI	54.35 (2.46)	74.41 (1.69)	64.94 (2.45)	78.31 (2.46)	10.591	**0.001**	20.054	** < 0.001**	−6.690	0.131
BRIEF MI	74.30 (3.27)	105.67 (1.81)	88.88 (3.75)	109.37 (2.21)	14.577	**0.002**	31.374	** < 0.001**	−10.883	0.054
BRIEF GEC	128.65 (5.53)	180.08 (3.15)	153.82 (5.92)	187.67 (4.35)	25.167	**0.001**	51.427	** < 0.001**	−17.573	0.063
BRIEF Negativity	0.72 (0.29)	2.18 (0.30)	1.70 (0.37)	3.32 (0.41)	0.987	**0.027**	1.462	** < 0.001**	0.155	0.815
BRIEF Inconsistency	4.37 (0.43)	5.96 (0.29)	5.65 (0.39)	6.25 (0.46)	1.271	**0.021**	1.585	**0.001**	−0.981	0.196
BRIEF Total	110.83 (4.68)	153.44 (2.74)	132.59 (4.95)	158.86 (3.85)	21.759	** < 0.001**	42.616	** < 0.001**	−16.343	**0.043**
**Panel B. Models Adjusted For Age (Months), Sex, BMI Z-Score, and CGI-S**										
SDQ Emotional Symptoms	4.87 (1.06)	2.72 (0.36)	4.20 (0.60)	5.28 (0.57)	−0.668	0.620	−2.148	0.078	3.227	**0.011**
SDQ Peer Problems	2.92 (0.88)	3.39 (0.34)	4.40 (0.59)	5.12 (0.59)	1.482	0.248	0.466	0.666	0.248	0.832
SDQ Conduct Problems	3.63 (1.06)	3.04 (0.40)	3.70 (0.63)	5.06 (0.66)	0.067	0.962	−0.592	0.652	1.954	0.167
SDQ Hyperactivity	4.44 (0.81)	6.60 (0.31)	3.76 (0.55)	5.11 (0.43)	−0.675	0.562	2.158	**0.024**	−0.809	0.470
SDQ Internalizing	7.79 (1.67)	6.11 (0.58)	8.60 (1.06)	10.40 (1.00)	0.814	0.725	−1.682	0.399	3.475	0.095
SDQ Externalizing	8.07 (1.53)	9.64 (0.54)	7.46 (1.00)	10.17 (0.83)	−0.608	0.770	1.566	0.387	1.145	0.570
SDQ Prosocial	8.39 (0.82)	7.49 (0.30)	7.22 (0.44)	6.92 (0.48)	−1.172	0.254	−0.898	0.344	0.601	0.561
SDQ Total Difficulties	15.86 (2.83)	15.74 (0.99)	16.07 (1.84)	20.57 (1.64)	0.206	0.957	−0.117	0.972	4.619	0.202
CPRS-Oppositional	6.73 (1.74)	9.19 (0.66)	7.69 (0.92)	9.94 (1.01)	0.957	0.663	2.459	0.222	−0.210	0.918
CPRS-Inattention	4.54 (1.52)	11.28 (0.60)	4.45 (1.00)	10.41 (0.86)	−0.093	0.961	6.736	** < 0.001**	−0.775	0.662
CPRS-Hyperactivity	4.75 (1.90)	9.43 (0.66)	5.76 (1.07)	10.53 (1.04)	1.016	0.675	4.682	**0.034**	0.089	0.970
CPRS-ADHD Index	10.42 (2.86)	21.38 (0.99)	12.14 (1.78)	20.93 (1.54)	1.711	0.644	10.959	** < 0.001**	−2.169	0.525
CPRS-Total Score	24.97 (6.64)	45.72 (2.10)	27.79 (3.74)	46.64 (3.63)	2.816	0.731	20.752	**0.006**	−1.902	0.800
NIAS Picky Eating	9.29 (1.62)	11.09 (0.71)	9.78 (0.97)	11.48 (0.73)	0.497	0.813	1.806	0.336	−0.108	0.960
NIAS Appetite	5.43 (1.75)	8.99 (0.67)	7.50 (0.98)	9.36 (0.99)	2.071	0.383	3.560	0.089	−1.699	0.421
NIAS Fear	5.28 (1.08)	5.21 (0.40)	3.87 (0.64)	7.00 (0.43)	−1.407	0.336	−0.072	0.952	3.196	**0.033**
NIAS Total	20.00 (3.06)	25.29 (1.34)	21.16 (1.82)	27.84 (1.39)	1.161	0.774	5.294	0.139	1.389	0.718
BRIEF Emotional Control	20.66 (2.20)	20.30 (0.69)	18.39 (1.35)	21.42 (1.57)	−2.275	0.469	−0.365	0.888	3.391	0.223
BRIEF Shift	18.41 (1.43)	21.79 (0.58)	19.91 (0.95)	22.83 (0.97)	1.506	0.457	3.385	**0.042**	−0.469	0.790
BRIEF Inhibit	24.84 (2.70)	30.64 (0.99)	23.28 (1.40)	29.56 (1.87)	−1.564	0.667	5.805	0.078	0.484	0.889
BRIEF Plan	24.45 (2.05)	31.31 (0.81)	25.25 (1.26)	31.97 (1.40)	0.801	0.773	6.865	**0.005**	−0.146	0.952
BRIEF Working Memory	19.99 (1.54)	23.54 (0.50)	18.31 (0.97)	22.61 (1.06)	−1.679	0.425	3.555	**0.043**	0.745	0.673
BRIEF Initiate	14.37 (1.12)	15.67 (0.40)	13.23 (0.76)	16.00 (0.62)	−1.140	0.467	1.301	0.315	1.468	0.310
BRIEF OFM	12.71 (1.55)	15.31 (0.55)	12.70 (0.94)	15.29 (1.07)	−0.011	0.996	2.597	0.151	−0.004	0.999
BRIEF Monitor	15.01 (1.21)	17.70 (0.42)	15.04 (0.70)	17.74 (0.71)	0.031	0.984	2.689	**0.047**	0.010	0.994
BRIEF BRI	63.91 (5.65)	72.74 (1.87)	61.58 (2.98)	73.81 (4.00)	−2.333	0.761	8.826	0.184	3.405	0.616
BRIEF MI	86.48 (6.08)	103.55 (1.99)	84.59 (3.85)	103.64 (4.13)	−1.889	0.819	17.068	**0.014**	1.979	0.779
BRIEF GEC	150.39 (10.99)	176.29 (3.40)	146.17 (6.34)	177.45 (7.72)	−4.222	0.775	25.893	**0.040**	5.384	0.670
BRIEF Negativity	1.71 (0.97)	2.00 (0.31)	1.35 (0.47)	2.85 (0.68)	−0.355	0.785	0.297	0.789	1.202	0.258
BRIEF Inconsistency	5.27 (0.90)	5.80 (0.30)	5.33 (0.44)	5.83 (0.58)	0.059	0.960	0.532	0.595	−0.034	0.974
BRIEF Total	129.04 (9.46)	150.27 (2.95)	126.18 (5.43)	150.30 (6.68)	−2.854	0.823	21.232	**0.050**	2.883	0.791

Results are based on 2 × 2 factorial linear regression models examining the main effects of encopresis (ENC: present vs. absent), attention-deficit/hyperactivity disorder (ADHD: present vs. absent), and their interaction (ENC × ADHD). Panel A presents models adjusted for age (months), sex, and BMI z-score. Panel B additionally includes clinician-rated global severity (CGI-S) as a covariate. Values are presented as estimated marginal means with standard errors (SE) for each group. Unstandardized regression coefficients (β) and corresponding *p* values are reported for main and interaction effects. Statistically significant effects (*p* < 0.05) are presented in bold. Given the number of statistical tests, false discovery rate (FDR) correction was applied to interaction terms; none of the interaction effects remained statistically significant after correction (see Supplementary Tables S1 and S2 for full models including robustness analyses). *SDQ* Strengths and Difficulties Questionnaire; *CPRS* Conners’ Parent Rating Scale; *NIAS* Nine Item Avoidant/Restrictive Food Intake Disorder Screen; *BRIEF* Behavior Rating Inventory of Executive Function; *BRI* Behavioral Regulation Index; *MI* Metacognition Index; *GEC* Global Executive Composite; *OFM* Organization of Materials

In Panel A, ENC showed significant main effects on several SDQ outcomes, including emotional symptoms (*β* = 1.851, *p* = 0.012), peer problems (*β* = 1.354, *p* = 0.018), conduct problems (*β* = 2.616, *p* < 0.001), internalizing problems (*β* = 3.205, *p* = 0.005), externalizing problems (*β* = 3.291, *p* = 0.002), and total difficulties (*β* = 6.496, *p* = 0.001), whereas ADHD showed significant main effects on hyperactivity (*β* = 3.331, *p* < 0.001), conduct problems (*β* = 1.622, *p* < 0.001), externalizing problems (*β* = 4.953, *p* < 0.001), and total difficulties (*β* = 5.348, *p* < 0.001). A significant ENC × ADHD interaction term was observed for SDQ hyperactivity (*β* = −1.863, *p* = 0.006), indicating that the joint effect of ENC and ADHD on hyperactivity was less than additive. However, this interaction did not remain significant after FDR correction in the supplementary robustness analyses. Across CPRS-R:S outcomes in Panel A, both ENC and ADHD showed significant main effects for oppositional symptoms, inattention, hyperactivity, ADHD Index, and total score, whereas significant ENC × ADHD interactions were also observed for oppositional symptoms (*β* = −2.895, *p* = 0.026), inattention (*β* = −2.943, *p* = 0.028), ADHD Index (*β* = −6.216, *p* = 0.008), and total score (*β* = −11.945, *p* = 0.021). In addition, NIAS Fear showed a significant ENC main effect (*β* = −1.684, *p* = 0.009) and a significant positive ENC × ADHD interaction (*β* = 3.412, *p* < 0.001), with the highest adjusted mean observed in the ENC + ADHD group. Among these interaction findings, only the NIAS Fear interaction remained significant after FDR correction and was also supported by permutation and bootstrap analyses (Supplementary Table S1). Within the BRIEF domain in Panel A, ENC and ADHD both showed broad main effects across multiple subscales and composite indices. Significant ENC main effects were observed for Emotional Control, Shift, Inhibit, Plan/Organize, Initiate, Organization of Materials, Monitor, BRI, MI, GEC, Negativity, Inconsistency, and Total Score (all *p* ≤ 0.032), whereas ADHD showed significant main effects across all BRIEF outcomes, including Emotional Control, Shift, Inhibit, Plan/Organize, Working Memory, Initiate, Organization of Materials, Monitor, BRI, MI, GEC, Negativity, Inconsistency, and Total Score (all *p* ≤ 0.001). Significant ENC × ADHD interactions emerged for BRIEF Monitor (*β* = −2.320, *p* = 0.028) and BRIEF Total Score (*β* = −16.343, *p* = 0.043), with near-significant interactions for Inhibit, MI, and GEC. However, none of the BRIEF interaction terms remained significant after FDR correction in the supplementary analyses.

After additional adjustment for CGI-S in Panel B, the overall pattern changed substantially. For SDQ outcomes, the previously observed ENC main effects were attenuated and no longer significant, whereas ADHD retained a significant main effect only for SDQ hyperactivity (*β* = 2.158, *p* = 0.024). A positive ENC × ADHD interaction emerged for SDQ emotional symptoms (*β* = 3.227, *p* = 0.011), suggesting higher emotional symptom levels in the comorbid group after accounting for overall clinical severity; however, this interaction did not remain significant after FDR correction. Across CPRS-R:S outcomes in Panel B, ADHD remained the dominant predictor, with significant main effects for inattention (*β* = 6.736, *p* < 0.001), hyperactivity (*β* = 4.682, *p* = 0.034), ADHD Index (*β* = 10.959, *p* < 0.001), and total score (*β* = 20.752, *p* = 0.006). In contrast, ENC main effects and all ENC × ADHD interaction terms for CPRS outcomes were no longer significant after CGI-S adjustment. The ENC × ADHD interaction for NIAS Fear remained nominally significant (*β* = 3.196, *p* = 0.033), again with the highest adjusted scores in the ENC + ADHD group. Nevertheless, this effect did not survive FDR correction in Supplementary Table S2. Within the BRIEF domain in Panel B, ADHD showed significant main effects on Shift (*β* = 3.385, *p* = 0.042), Plan/Organize (*β* = 6.865, *p* = 0.005), Working Memory (*β* = 3.555, *p* = 0.043), Monitor (*β* = 2.689, *p* = 0.047), MI (*β* = 17.068, *p* = 0.014), and GEC (*β* = 25.893, *p* = 0.040), with a borderline effect for BRIEF Total Score (*β* = 21.232, *p* = 0.050). ENC showed no significant main effects across BRIEF outcomes, and no ENC × ADHD interaction terms were significant. Validity-related BRIEF scales also showed no significant main or interaction effects after CGI-S adjustment.

## Discussion

To our knowledge, this is the first multicenter cross-sectional study to examine the independent and interactive associations of ADHD and ENC with internalizing and externalizing symptoms, executive functioning, and ADHD- and ARFID-related symptoms in early childhood. Overall, the findings indicate that the co-occurrence of ENC and ADHD is associated with the most adverse clinical profile, including greater overall symptom severity, more pronounced executive-functioning difficulties, higher levels of emotional–behavioral problems, and greater fear-based eating symptoms compared with either condition alone. Within the factorial analytic models, ADHD showed consistent main associations with executive-functioning difficulties and core ADHD symptom dimensions, whereas ENC-specific associations were more evident in internalizing symptoms and toileting-related severity. In addition, ENC × ADHD interaction terms were observed for selected outcomes, particularly emotional symptoms and fear-based eating, suggesting that comorbidity may be linked to a clinical burden that is not fully captured by simple groupwise additivity alone.

The high incidence of co-occurrence of ADHD and ENC is well documented in the literature (McKeown et al., 2013; Mohammadi et al., 2021; Unal & Pehlivantürk, 2004). Consistent with this, a large-scale, population-based study demonstrated that children with ENC were significantly more likely to have ADHD than those with other functional urination and defecation disorders. This co-occurrence is thought to be linked to both behavioral processes and possible shared neurobiological mechanisms, involving impaired integration between the central nervous system and the enteric nervous system (McKeown et al., 2013). Supporting this perspective, a study involving children aged 7–15 years diagnosed with ENC and comorbid ADHD reported significant reductions in both core ADHD symptoms and the frequency of ENC following long-acting methylphenidate treatment (Yilmaz et al., 2014). It has been suggested that the effects of methylphenidate on ENC may be related to improvements in executive functioning, particularly self-regulation, impulse control, and self-awareness components, as well as its regulatory effects on gastrointestinal motility via dopaminergic receptors (Golubchik & Weizman, 2009; Huang et al., 2012). Taken together with the high comorbidity rates, these findings are consistent with the possibility that ENC and ADHD may involve overlapping clinical correlates or partially overlapping processes. Accordingly, when ENC and ADHD co-occur, the clinical profile may be more severe across multiple clinical dimensions. Indeed, the significant ENC × ADHD interaction found in our study for emotional symptoms and eating-related fear provides additional support for an increased clinical burden when these two conditions co-occur. On the other hand, the same study found that a reduction in the frequency of ENC was not linked to an improvement in the core symptoms of ADHD (Yilmaz et al., 2014). This suggests that, despite shared mechanisms between ENC and ADHD, the two clinical conditions may also differ in certain aspects. Therefore, it is important to consider both overlapping and disorder-specific clinical features in our understanding and clinical management of these conditions.

Although the effects of ADHD on core symptoms and executive functioning are well defined in the literature, another unique contribution of this study is the identification of ENC-specific findings that are distinctive and clinically informative. Consistent with our findings, a meta-analysis revealed that elimination disorders are associated with higher levels of internalizing symptoms (Aymerich et al., 2023). In addition, another study found that constipation is closely related to increased behavioral and emotional problems, regardless of fecal incontinence (dos Santos et al., 2021). It has been suggested that chronic constipation and ENC may negatively affect emotion regulation processes through increased visceral sensitivity and overactivation of stress response systems, thereby creating a vulnerability to internalizing symptoms (Chitkara et al., 2008). The same meta-analysis also demonstrated an association between elimination disorders and impaired self-concept and low self-esteem, which are well-established psychological risk factors for internalizing symptoms (Aymerich et al., 2023). On the other hand, adverse childhood events, a significant area of research in the etiology of ENC, are considered a common risk factor for both ENC and internalizing symptoms (Briggs-Gowan et al., 2010; Melchior et al., 2014; Philips et al., 2015). Indeed, our finding of higher levels of adverse childhood events in the ENC group is consistent with previous research (Foreman & Thambirajah, 1996). Early stressors have been reported to lead to increased sensitivity in stress response systems, which may be associated with the emergence of unexplained gastrointestinal symptoms (Grasso et al., 2013; Philips et al., 2015). In addition, early adverse experiences may also contribute to the onset and maintenance of elimination symptoms through elevated levels of psychopathology (Hobbis et al., 2002). However, due to the cross-sectional design of our study, the direction of the relationships between internalizing symptoms, adverse childhood events, and ENC cannot be established and remains unclear in the existing literature. In addition, the increased levels of food-related fear observed in the ENC group may represent another ENC-specific manifestation of internalizing vulnerability. Food-related fear may reflect heightened interoceptive hypersensitivity (Zickgraf & Elkins, 2018). Although sensory processing patterns were not directly examined in the present study, a limited number of studies have reported sensory processing abnormalities and sensory overresponsivity in children with functional defecation disorders, lending support to this interpretation (Beaudry-Bellefeuille & Lane, 2017; Beaudry-Bellefeuille et al., 2019). Despite the clinical overlap between ADHD and ENC, the present findings suggest that ENC may be associated with a relatively distinct profile of internalizing difficulties, including anxiety/depression symptoms, adverse childhood events, and food-related fears. Therefore, the clinical assessment of children with ENC should be conducted within a broader psychosocial and emotional context, and these components should be integrated into intervention programs.

Our findings showed that retentive ENC was more prevalent in the ENC + ADHD group compared to the ENC-only group, and that the ENC + ADHD group exhibited more severe constipation and toileting-related symptoms. This pattern may be associated with ADHD-specific executive functioning difficulties, including impaired recognition of toileting signals, difficulty disengaging from ongoing play or leisure activities, procrastination, and difficulties in maintaining eating and toileting routines, all of which may contribute to delayed defecation (Liu & Zhang, 2022; Rey & Omigbodun, 2015). Furthermore, considering the higher levels of internalizing and externalizing symptoms in this group, conditioned avoidance of toileting behavior following repeated unsuccessful toilet training experiences, as well as increased refusal to go to the toilet —particularly in the context of parent–child conflict—may further reinforce fecal retention (Fishman et al., 2002; Nurko & Scott, 2011). These findings highlight the importance of a multifaceted approach to evaluate constipation and toileting-related symptoms, particularly in children with ENC and comorbid ADHD. The subtype findings main contribution is to highlight heterogeneity within the encopresis phenotype, particularly given that retentive and non-retentive forms differ in clinical presentation and may have partly distinct diagnostic and management implications.

### Strengths and Limitations

To the best of our knowledge, this is the first study to examine the independent and interactive associations of ENC and ADHD. The key strengths of the study include its four-group design, the inclusion of drug-naïve cases, the formation of groups based on diagnoses established through clinician-administered semi-structured interviews, and the detailed assessment of sociodemographic and clinical characteristics, as well as toileting-related variables. In addition, the holistic evaluation of comorbid internalizing and externalizing psychiatric symptoms, executive functioning, and ADHD- and ARFID-related symptoms, together with the examination of these domains using factorial regression analyses with covariate control, strengthens the reliability of the findings. However, several limitations should be considered, including the cross-sectional design, the relatively small sample size, reliance on parental-report measures. Because ADHD-related symptoms, emotional-behavioral outcomes, executive functioning, and eating-related features were assessed primarily through the same informant source, some of the observed associations may have been influenced by shared method variance. This issue may be particularly relevant to the association between ADHD-related symptoms and BRIEF-based executive-function ratings. In addition, the absence of performance-based neuropsychological measures limits conclusions regarding the specificity of the executive-function findings; accordingly, the BRIEF results should be interpreted as reflecting ecologically rated executive difficulties in everyday life rather than performance-based executive functioning per se. Because the ADHD groups were composed of newly diagnosed, pre-treatment cases, the findings may not be fully generalizable to treated or more chronic clinical ADHD samples. Furthermore, as the study was conducted in a clinical sample, the findings may not be generalizable to the general population. Socioeconomic status was not assessed using a structured measure, which limits interpretation of contextual factors, particularly with respect to psychosocial stressors. In addition, because the study was cross-sectional and observational, the findings cannot be used to infer shared etiological mechanisms, mediational pathways, or synergistic developmental processes; the interaction terms should be interpreted as statistical interaction within this dataset rather than evidence of causal interaction. The fear-related eating findings observed in the present study should be interpreted cautiously. Rather than implying that eating-related symptoms constitute a core feature of ENC-ADHD comorbidity, our results suggest that they may reflect one clinically relevant and relatively understudied domain within a broader multidimensional self-regulatory profile.

## Conclusion and Future Directions

Although ENC and ADHD frequently co-occur and show some overlapping clinical correlates, they also appear to have partially distinct clinical profiles Our findings suggest that managing ENC solely as an isolated gastrointestinal problem may limit effective clinical management, particularly in the presence of comorbid ADHD. To effectively manage children with ENC and comorbid ADHD, it is crucial to support executive functioning, appropriately address comorbid psychiatric symptoms, and target eating-related fears as fundamental components of a holistic intervention. Furthermore, our findings suggest that ENC may be associated with a distinct profile of internalizing vulnerability, emphasizing the importance of comprehensive evaluation of these children with regard to internalizing symptoms, adverse childhood events, and eating-related fears. Future longitudinal and intervention-focused studies would be important in revealing the long-term clinical outcomes of this holistic approach, as well as identifying which subgroups benefit most from which interventions.

## Electronic Supplementary Material

Below is the link to the electronic supplementary material.

Supplementary Materials

## Data Availability

The datasets analyzed in the current study are available from the corresponding author upon reasonable request.
