# Supplementary material for: Independent and Interactive Associations of Encopresis and Attention-Deficit/Hyperactivity Disorder in Children on Emotional–Behavioral Symptoms, Executive Functioning, and Avoidant/Restrictive Food Intake–Related Symptoms
Source: Res Child Adolesc Psychopathol. 2026 Apr 21;54(3):64. doi: 10.1007/s10802-026-01463-w (PMC13099798; doi:10.1007/s10802-026-01463-w)
Supplement: Supplementary file 1 — Supplementary Materials [file 10802_2026_1463_MOESM1_ESM.docx]

**SUPPLEMANTARY MATERIALS**

**Table S1**

Interaction models for all outcomes (Sex, age, BMI z-score adjusted)

| **Outcome** | **ENC β** | **ENC p (HC3)** | **ADHD β** | **ADHD p (HC3)** | **Interaction β** | **Interaction SE** | **Interaction t** | **Interaction p (HC3)** | **Interaction 95% CI** | **Interaction p (perm)** | **Bootstrap median** | **Bootstrap 95% CI** | **R²** | **Adj. R²** | **Interaction FDR p (HC3)** | **Interaction FDR p (perm)** |
| --- | --- | --- | --- | --- | --- | --- | --- | --- | --- | --- | --- | --- | --- | --- | --- | --- |
| SDQ Emotional Symptoms | 1.851 | **0.012** | 0.041 | 0.944 | 1.259 | 0.905 | 1.390 | 0.165 | [-0.52, 3.03] | 0.148 | 1.278 | [-0.49, 3.06] | 0.245 | 0.212 | 0.268 | 0.242 |
| SDQ Peer Problems | 1.354 | **0.018** | 0.355 | 0.450 | 0.348 | 0.736 | 0.473 | 0.636 | [-1.09, 1.79] | 0.625 | 0.284 | [-1.08, 1.72] | 0.167 | 0.132 | 0.822 | 0.807 |
| SDQ Conduct Problems | 2.616 | **<0.001** | 1.622 | **<0.001** | -0.037 | 0.877 | -0.043 | 0.966 | [-1.76, 1.68] | 0.966 | -0.018 | [-1.71, 1.70] | 0.288 | 0.258 | 0.966 | 0.966 |
| SDQ Hyperactivity | 0.675 | 0.217 | 3.331 | **<0.001** | -1.863 | 0.680 | -2.741 | **0.006** | [-3.20, -0.53] | **0.003** | -1.888 | [-3.17, -0.58] | 0.326 | 0.297 | 0.078 | **0.046** |
| SDQ Internalizing | 3.205 | **0.005** | 0.395 | 0.645 | 1.606 | 1.380 | 1.164 | 0.245 | [-1.10, 4.31] | 0.242 | 1.643 | [-1.01, 4.27] | 0.272 | 0.241 | 0.345 | 0.341 |
| SDQ Externalizing | 3.291 | **0.002** | 4.953 | **<0.001** | -1.901 | 1.282 | -1.482 | 0.138 | [-4.41, 0.61] | 0.119 | -1.900 | [-4.41, 0.59] | 0.300 | 0.270 | 0.267 | 0.242 |
| SDQ Prosocial | -0.647 | 0.192 | -0.442 | 0.292 | 0.191 | 0.696 | 0.275 | 0.784 | [-1.17, 1.55] | 0.778 | 0.193 | [-1.17, 1.48] | 0.043 | 0.002 | 0.890 | 0.890 |
| SDQ Total Difficulties | 6.496 | **0.001** | 5.348 | **<0.001** | -0.294 | 2.429 | -0.121 | 0.904 | [-5.05, 4.47] | 0.904 | -0.331 | [-4.84, 4.13] | 0.279 | 0.249 | 0.934 | 0.934 |
| CPRS-Oppositional | 4.393 | **<0.001** | 5.445 | **<0.001** | -2.895 | 1.304 | -2.220 | **0.026** | [-5.45, -0.34] | **0.025** | -2.931 | [-5.23, -0.45] | 0.296 | 0.266 | 0.124 | 0.122 |
| CPRS-Inattention | 2.683 | **0.014** | 9.148 | **<0.001** | -2.943 | 1.339 | -2.198 | **0.028** | [-5.57, -0.32] | **0.023** | -2.960 | [-5.75, -0.39] | 0.545 | 0.526 | 0.124 | 0.122 |
| CPRS-Hyperactivity | 3.001 | **0.009** | 6.407 | **<0.001** | -1.462 | 1.507 | -0.970 | 0.332 | [-4.42, 1.49] | 0.330 | -1.467 | [-4.42, 1.43] | 0.331 | 0.303 | 0.448 | 0.445 |
| CPRS-ADHD Index | 6.893 | **<0.001** | 15.461 | **<0.001** | -6.216 | 2.326 | -2.672 | 0.008 | [-10.78, -1.66] | **0.007** | -6.268 | [-10.64, -1.64] | 0.516 | 0.495 | 0.078 | 0.077 |
| CPRS-Total Score | 15.674 | **<0.001** | 31.923 | **<0.001** | -11.945 | 5.176 | -2.308 | 0.021 | [-22.09, -1.80] | **0.017** | -11.966 | [-22.19, -2.16] | 0.484 | 0.462 | 0.124 | 0.122 |
| NIAS Picky Eating | 0.736 | 0.502 | 2.013 | 0.063 | -0.295 | 1.398 | -0.211 | 0.833 | [-3.03, 2.44] | 0.844 | -0.309 | [-2.88, 2.43] | 0.070 | 0.030 | 0.890 | 0.902 |
| NIAS Appetite | -0.775 | 0.478 | 1.087 | 0.274 | 0.524 | 1.375 | 0.381 | 0.703 | [-2.17, 3.22] | 0.695 | 0.470 | [-2.09, 3.06] | 0.139 | 0.103 | 0.872 | 0.862 |
| NIAS Fear | -1.684 | **0.009** | -0.312 | 0.665 | 3.412 | 0.855 | 3.989 | **<0.001** | [1.74, 5.09] | **<0.001** | 3.422 | [1.83, 5.04] | 0.115 | 0.077 | **0.002** | **0.015** |
| NIAS Total | -1.723 | 0.418 | 2.789 | 0.201 | 3.642 | 2.562 | 1.422 | 0.155 | [-1.38, 8.66] | 0.134 | 3.608 | [-1.43, 8.60] | 0.105 | 0.067 | 0.267 | 0.242 |
| BRIEF Emotional Control | 2.745 | **0.032** | 3.998 | **<0.001** | -0.531 | 1.755 | -0.303 | 0.762 | [-3.97, 2.91] | 0.776 | -0.581 | [-3.98, 2.67] | 0.162 | 0.126 | 0.890 | 0.890 |
| BRIEF Shift | 3.647 | **<0.001** | 5.245 | **<0.001** | -2.142 | 1.293 | -1.656 | 0.098 | [-4.68, 0.39] | 0.093 | -2.131 | [-4.57, 0.46] | 0.307 | 0.277 | 0.233 | 0.223 |
| BRIEF Inhibit | 4.198 | **0.004** | 10.811 | **<0.001** | -4.017 | 2.109 | -1.905 | 0.057 | [-8.15, 0.12] | **0.050** | -4.050 | [-8.11, -0.29] | 0.389 | 0.363 | 0.176 | 0.174 |
| BRIEF Plan | 4.471 | **0.002** | 10.054 | **<0.001** | -3.013 | 1.811 | -1.663 | 0.096 | [-6.56, 0.54] | 0.080 | -3.087 | [-6.62, 0.37] | 0.453 | 0.430 | 0.233 | 0.207 |
| BRIEF Working Memory | 1.970 | 0.105 | 6.724 | **<0.001** | -2.105 | 1.452 | -1.449 | 0.147 | [-4.95, 0.74] | 0.141 | -2.189 | [-4.84, 0.66] | 0.398 | 0.372 | 0.267 | 0.242 |
| BRIEF Initiate | 2.466 | **0.004** | 4.434 | **<0.001** | -1.349 | 1.039 | -1.298 | 0.194 | [-3.39, 0.69] | 0.189 | -1.310 | [-3.41, 0.64] | 0.347 | 0.319 | 0.290 | 0.279 |
| BRIEF OFM | 2.623 | **0.008** | 4.885 | **<0.001** | -2.061 | 1.403 | -1.468 | 0.142 | [-4.81, 0.69] | 0.135 | -2.070 | [-4.69, 0.72] | 0.253 | 0.221 | 0.267 | 0.242 |
| BRIEF Monitor | 3.014 | **<0.001** | 5.281 | **<0.001** | -2.320 | 1.056 | -2.196 | **0.028** | [-4.39, -0.25] | **0.027** | -2.397 | [-4.35, -0.36] | 0.394 | 0.368 | 0.124 | 0.122 |
| BRIEF BRI | 10.591 | **0.001** | 20.054 | **<0.001** | -6.690 | 4.426 | -1.511 | 0.131 | [-15.36, 1.99] | 0.125 | -6.680 | [-15.13, 1.60] | 0.349 | 0.321 | 0.267 | 0.242 |
| BRIEF MI | 14.577 | **0.002** | 31.374 | **<0.001** | -10.883 | 5.639 | -1.930 | 0.054 | [-21.94, 0.17] | 0.057 | -11.153 | [-21.88, -0.32] | 0.475 | 0.452 | 0.176 | 0.178 |
| BRIEF GEC | 25.167 | **0.001** | 51.427 | **<0.001** | -17.573 | 9.463 | -1.857 | 0.063 | [-36.12, 0.97] | 0.073 | -17.582 | [-35.43, 0.06] | 0.452 | 0.429 | 0.178 | 0.206 |
| BRIEF Negativity | 0.987 | **0.027** | 1.462 | **<0.001** | 0.155 | 0.662 | 0.234 | 0.815 | [-1.14, 1.45] | 0.804 | 0.165 | [-1.02, 1.40] | 0.159 | 0.123 | 0.890 | 0.890 |
| BRIEF Inconsistency | 1.271 | **0.021** | 1.585 | **0.001** | -0.981 | 0.760 | -1.292 | 0.196 | [-2.47, 0.51] | 0.178 | -0.950 | [-2.38, 0.46] | 0.120 | 0.082 | 0.290 | 0.276 |
| BRIEF Total | 21.759 | **<0.001** | 42.616 | **<0.001** | -16.343 | 8.066 | -2.026 | **0.043** | [-32.15, -0.53] | **0.033** | -17.112 | [-31.00, -0.71] | 0.431 | 0.406 | 0.166 | 0.130 |

**Note.** Results are based on 2 × 2 factorial linear regression models examining the main effects of encopresis (ENC: present vs. absent), attention-deficit/hyperactivity disorder (ADHD: present vs. absent), and their interaction (ENC × ADHD). Table S1 presents models adjusted for age (months), sex, and BMI z-score. Unstandardized regression coefficients (β), heteroscedasticity-consistent (HC3) robust standard errors, t values, and corresponding p values are reported. To evaluate the robustness of interaction effects, permutation-based p values and bootstrap estimates (median and 95% confidence intervals) are provided. Model fit is summarized using R² and adjusted R². Given the large number of statistical tests, false discovery rate (FDR) correction was applied to interaction terms based on both HC3 and permutation-derived p values. SDQ = Strengths and Difficulties Questionnaire; CPRS = Conners’ Parent Rating Scale; NIAS = Nine Item Avoidant/Restrictive Food Intake Disorder Screen; BRIEF = Behavior Rating Inventory of Executive Function; BRI = Behavioral Regulation Index; MI = Metacognition Index; GEC = Global Executive Composite; OFM = Organization of Materials.

**Table S2**

Interaction models for all outcomes (Sex, age, BMI z-score, CGI-S adjusted)

| **Outcome** | **ENC β** | **ENC p (HC3)** | **ADHD β** | **ADHD p (HC3)** | **Interaction β** | **Interaction SE** | **Interaction t** | **Interaction p (HC3)** | **Interaction 95% CI** | **Interaction p (perm)** | **Bootstrap median** | **Bootstrap 95% CI** | **R²** | **Adj. R²** | **Interaction FDR p (HC3)** | **Interaction FDR p (perm)** |
| --- | --- | --- | --- | --- | --- | --- | --- | --- | --- | --- | --- | --- | --- | --- | --- | --- |
| SDQ Emotional Symptoms | -0.668 | 0.620 | -2.148 | 0.078 | 3.227 | 1.275 | 2.530 | **0.011** | [0.73, 5.73] | **0.011** | 3.183 | [0.79, 5.65] | 0.271 | 0.235 | 0.354 | 0.341 |
| SDQ Peer Problems | 1.482 | 0.248 | 0.466 | 0.666 | 0.248 | 1.167 | 0.212 | 0.832 | [-2.04, 2.53] | 0.824 | 0.195 | [-1.95, 2.31] | 0.167 | 0.126 | 0.999 | 0.999 |
| SDQ Conduct Problems | 0.067 | 0.962 | -0.592 | 0.652 | 1.954 | 1.413 | 1.382 | 0.167 | [-0.82, 4.72] | 0.159 | 1.991 | [-0.65, 4.88] | 0.314 | 0.280 | 0.999 | 0.999 |
| SDQ Hyperactivity | -0.675 | 0.562 | 2.158 | **0.024** | -0.809 | 1.119 | -0.723 | 0.470 | [-3.00, 1.38] | 0.458 | -0.853 | [-2.90, 1.27] | 0.335 | 0.302 | 0.999 | 0.999 |
| SDQ Internalizing | 0.814 | 0.725 | -1.682 | 0.399 | 3.475 | 2.079 | 1.672 | 0.095 | [-0.60, 7.55] | 0.084 | 3.512 | [-0.51, 7.59] | 0.282 | 0.246 | 0.978 | 0.873 |
| SDQ Externalizing | -0.608 | 0.770 | 1.566 | 0.387 | 1.145 | 2.013 | 0.568 | 0.570 | [-2.80, 5.09] | 0.541 | 1.136 | [-2.47, 5.19] | 0.326 | 0.292 | 0.999 | 0.999 |
| SDQ Prosocial | -1.172 | 0.254 | -0.898 | 0.344 | 0.601 | 1.033 | 0.582 | 0.561 | [-1.42, 2.63] | 0.545 | 0.594 | [-1.25, 2.56] | 0.045 | -0.003 | 0.999 | 0.999 |
| SDQ Total Difficulties | 0.206 | 0.957 | -0.117 | 0.972 | 4.619 | 3.622 | 1.275 | 0.202 | [-2.48, 11.72] | 0.196 | 4.428 | [-2.29, 11.92] | 0.301 | 0.266 | 0.999 | 0.999 |
| CPRS-Oppositional | 0.957 | 0.663 | 2.459 | 0.222 | -0.210 | 2.035 | -0.103 | 0.918 | [-4.20, 3.78] | 0.919 | -0.186 | [-3.81, 3.89] | 0.313 | 0.278 | 0.999 | 0.999 |
| CPRS-Inattention | -0.093 | 0.961 | 6.736 | **<0.001** | -0.775 | 1.773 | -0.437 | 0.662 | [-4.25, 2.70] | 0.651 | -0.733 | [-4.32, 2.50] | 0.553 | 0.531 | 0.999 | 0.999 |
| CPRS-Hyperactivity | 1.016 | 0.675 | 4.682 | **0.034** | 0.089 | 2.376 | 0.037 | 0.970 | [-4.57, 4.75] | 0.968 | 0.057 | [-4.12, 4.85] | 0.336 | 0.303 | 0.999 | 0.999 |
| CPRS-ADHD Index | 1.711 | 0.644 | 10.959 | **<0.001** | -2.169 | 3.415 | -0.635 | 0.525 | [-8.86, 4.52] | 0.515 | -2.107 | [-8.79, 4.51] | 0.526 | 0.502 | 0.999 | 0.999 |
| CPRS-Total Score | 2.816 | 0.731 | 20.752 | **0.006** | -1.902 | 7.522 | -0.253 | 0.800 | [-16.64, 12.84] | 0.785 | -2.126 | [-16.31, 12.75] | 0.497 | 0.471 | 0.999 | 0.999 |
| NIAS Picky Eating | 0.497 | 0.813 | 1.806 | 0.336 | -0.108 | 2.145 | -0.050 | 0.960 | [-4.31, 4.10] | 0.956 | -0.138 | [-3.89, 4.17] | 0.070 | 0.023 | 0.999 | 0.999 |
| NIAS Appetite | 2.071 | 0.383 | 3.560 | 0.089 | -1.699 | 2.111 | -0.805 | 0.421 | [-5.84, 2.44] | 0.410 | -1.778 | [-5.63, 2.35] | 0.153 | 0.111 | 0.999 | 0.999 |
| NIAS Fear | -1.407 | 0.336 | -0.072 | 0.952 | 3.196 | 1.498 | 2.134 | **0.033** | [0.26, 6.13] | **0.027** | 3.132 | [0.41, 5.94] | 0.115 | 0.071 | 0.509 | 0.418 |
| NIAS Total | 1.161 | 0.774 | 5.294 | 0.139 | 1.389 | 3.840 | 0.362 | 0.718 | [-6.14, 8.91] | 0.706 | 1.550 | [-6.00, 9.28] | 0.109 | 0.064 | 0.999 | 0.999 |
| BRIEF Emotional Control | -2.275 | 0.469 | -0.365 | 0.888 | 3.391 | 2.782 | 1.219 | 0.223 | [-2.06, 8.84] | 0.221 | 3.490 | [-1.99, 8.41] | 0.192 | 0.151 | 0.999 | 0.999 |
| BRIEF Shift | 1.506 | 0.457 | 3.385 | **0.042** | -0.469 | 1.762 | -0.266 | 0.790 | [-3.92, 2.98] | 0.788 | -0.411 | [-3.66, 3.05] | 0.314 | 0.280 | 0.999 | 0.999 |
| BRIEF Inhibit | -1.564 | 0.667 | 5.805 | 0.078 | 0.484 | 3.472 | 0.139 | 0.889 | [-6.32, 7.29] | 0.888 | 0.601 | [-5.59, 7.13] | 0.408 | 0.378 | 0.999 | 0.999 |
| BRIEF Plan | 0.801 | 0.773 | 6.865 | **0.005** | -0.146 | 2.399 | -0.061 | 0.952 | [-4.85, 4.56] | 0.952 | -0.105 | [-4.83, 4.32] | 0.462 | 0.436 | 0.999 | 0.999 |
| BRIEF Working Memory | -1.679 | 0.425 | 3.555 | **0.043** | 0.745 | 1.763 | 0.423 | 0.673 | [-2.71, 4.20] | 0.667 | 0.693 | [-2.66, 4.17] | 0.416 | 0.387 | 0.999 | 0.999 |
| BRIEF Initiate | -1.140 | 0.467 | 1.301 | 0.315 | 1.468 | 1.446 | 1.015 | 0.310 | [-1.37, 4.30] | 0.276 | 1.438 | [-1.28, 4.21] | 0.380 | 0.349 | 0.999 | 0.999 |
| BRIEF OFM | -0.011 | 0.996 | 2.597 | 0.151 | -0.004 | 2.002 | -0.002 | 0.999 | [-3.93, 3.92] | 0.999 | -0.003 | [-3.80, 3.98] | 0.265 | 0.229 | 0.999 | 0.999 |
| BRIEF Monitor | 0.031 | 0.984 | 2.689 | **0.047** | 0.010 | 1.421 | 0.007 | 0.994 | [-2.77, 2.80] | 0.994 | -0.082 | [-2.70, 2.75] | 0.413 | 0.384 | 0.999 | 0.999 |
| BRIEF BRI | -2.333 | 0.761 | 8.826 | 0.184 | 3.405 | 6.783 | 0.502 | 0.616 | [-9.89, 16.70] | 0.622 | 3.547 | [-9.21, 16.05] | 0.371 | 0.340 | 0.999 | 0.999 |
| BRIEF MI | -1.889 | 0.819 | 17.068 | **0.014** | 1.979 | 7.060 | 0.280 | 0.779 | [-11.86, 15.82] | 0.791 | 1.799 | [-11.99, 15.43] | 0.495 | 0.470 | 0.999 | 0.999 |
| BRIEF GEC | -4.222 | 0.775 | 25.893 | **0.040** | 5.384 | 12.617 | 0.427 | 0.670 | [-19.34, 30.11] | 0.660 | 5.697 | [-17.57, 28.87] | 0.476 | 0.449 | 0.999 | 0.999 |
| BRIEF Negativity | -0.355 | 0.785 | 0.297 | 0.789 | 1.202 | 1.064 | 1.131 | 0.258 | [-0.88, 3.29] | 0.257 | 1.251 | [-0.70, 3.28] | 0.172 | 0.130 | 0.999 | 0.999 |
| BRIEF Inconsistency | 0.059 | 0.960 | 0.532 | 0.595 | -0.034 | 1.070 | -0.032 | 0.974 | [-2.13, 2.06] | 0.982 | -0.038 | [-2.01, 2.09] | 0.128 | 0.085 | 0.999 | 0.999 |
| BRIEF Total | -2.854 | 0.823 | 21.232 | **0.050** | 2.883 | 10.882 | 0.265 | 0.791 | [-18.44, 24.21] | 0.778 | 3.015 | [-17.22, 22.88] | 0.454 | 0.427 | 0.999 | 0.999 |

**Note.** Results are based on 2 × 2 factorial linear regression models examining the main effects of encopresis (ENC: present vs. absent), attention-deficit/hyperactivity disorder (ADHD: present vs. absent), and their interaction (ENC × ADHD). Table S2 presents models adjusted for age (months), sex, BMI z-score, and clinician-rated global severity (CGI-S). Unstandardized regression coefficients (β), heteroscedasticity-consistent (HC3) robust standard errors, t values, and corresponding p values are reported. To evaluate the robustness of interaction effects, permutation-based p values and bootstrap estimates (median and 95% confidence intervals) are provided. Model fit is summarized using R² and adjusted R². Given the large number of statistical tests, false discovery rate (FDR) correction was applied to interaction terms based on both HC3 and permutation-derived p values. SDQ = Strengths and Difficulties Questionnaire; CPRS = Conners’ Parent Rating Scale; NIAS = Nine Item Avoidant/Restrictive Food Intake Disorder Screen; BRIEF = Behavior Rating Inventory of Executive Function; BRI = Behavioral Regulation Index; MI = Metacognition Index; GEC = Global Executive Composite; OFM = Organization of Materials.
